# Advances and Challenges in Pulsed Lasers Based on Low-Dimensional Material Saturable Absorbers

**DOI:** 10.3390/nano16130819

**Published:** 2026-07-02

**Authors:** Wenpei Zhang, Haotian Lu, Yunrou Wu, Weitao Liu, Tinglun Xing, Xi Wang, Xin Zhang, Ke Chen

**Affiliations:** 1 Center for the Physics of Low-Dimensional Materials, Henan Key Laboratory of Quantum Materials and Quantum Energy, School of Future Technology, Henan University, Kaifeng 475004, China; 2 International Joint Research Laboratory of New Energy Materials and Devices of Henan Province, School of Physics and Electronics, Henan University, Kaifeng 475400, China; 3 Photoelectric Countermeasure Department, Changchun Institute of Optics, Fine Mechanics and Physics, Chinese Academy of Sciences, Changchun 130033, China

**Keywords:** low-dimensional materials, saturable absorbers, pulsed laser, passive Q-switching, passive mode-locking

## Abstract

Low-dimensional materials (LDMs) are often favored by researchers in the ultrafast photonics field for their low optical loss, ultrafast carrier response, broadband nonlinear absorption, and easy integration with optoelectronic systems. High-performance broadband saturable absorbers (SAs) fabricated from LDMs have become core components for achieving compact and miniaturized ultrafast laser. This paper systematically reviews the laser applications of LDM SAs in the near/mid-infrared spectral region, focusing on the pulse modulation mechanisms, material systems, and device integration approaches. It analyzes current research progress and challenges while outlining future development trends for LDM SAs in ultrafast pulsed lasers and optoelectronic devices.

## 1. Introduction

Laser technology is critical to the development of modern science, industry, medicine, and communications [1,2,3]. Pulsed lasers operating at nanosecond, picosecond, and even femtosecond scales emit a significant quantity of energy within an ultrashort time interval that leads to their giant pulse energy and ultra-high peak power. They serve as indispensable instruments in domains such as ultrafast process analysis, optical storage, quantum computing, and micro-nano machining [4,5,6,7]. Typical types of lasers encompass solid-state lasers [7], optical parametric amplifiers [8], semiconductor lasers [9], and fiber lasers [10]. Laser pulsed modulation techniques can be classified into active modulation (e.g., electro-optic modulation [11], acousto-optic modulation [12], Mach-Zehnder modulators [13]) and passive modulation (e.g., saturable absorbers [14] and nonlinear polarization rotation [15]). Among them, passive Q-switching (QS) and mode-locking (ML) technologies based on saturable absorbers (SAs) have attracted much attention, as theirs structural simplicity, fast response, and low cost [16,17,18]. Exploring novel saturable absorbers (SAs) with high-performance is of great interest for compact and miniaturized commercial ultrafast pulsed lasers.

Early SAs include organic dyes [19], transition-metal-ion-doped crystals [20], color-center crystals [21], and semiconductor saturable absorber mirrors (SESAMs) [22]. The poor thermal stability of organic dyes and the long lifetime of transition-metal-ion-doped crystals limit their practical usage. SESAMs consist of a Bragg mirror and a semiconductor absorption layer (composed of III-V group compounds such as AlGaAs, InGaAs, or InAlAs). The saturable absorption characteristics can be realized through the adjustment of the thickness of the semiconductor absorption material and the quantity of Bragg mirror layers [23,24,25]. SESAMs are commonly used in various mode-locked lasers for their stable operation and high reproducibility, but they still exhibit wavelength sensitivity resulting from narrow nonlinear optical bandwidths and sophisticated fabrication processes. In recent years, a series of low-dimensional nanomaterials (LDMs), including graphene [26], black phosphorus (BP) [27], transition metal dichalcogenides (TMDs) [28,29,30], MXenes [31], carbon nanotubes (CNTs) [32], transition metal oxides (TMOs) [33], topological insulators (TIs) [34], metal-organic frameworks (MOFs) [35], and covalent organic frameworks (COFs) [36], have demonstrated advantages such as ultrafast carrier response, broadband modulation, low insertion loss, and tunable layered structures, spawning a series of innovative applications in ultrafast photonics. With the development of the integration processes with laser devices, the problems of stability and damage threshold of LDMs are continuously being improved, significantly driving the enhancement of laser performance.

This paper, beginning with an overview of passive QS and ML technologies based on SAs, reviews recent progress on LDM SAs, including graphene, BP, TMDs, heterostructures, and so on. Their laser applications in the near/mid-infrared spectral region is emphatically discussed. Furthermore, the novel advances on synthesis methods of LDM SAs are explored. This review finally summarizes the challenges associated with the future development of LDM SAs, which provides an outlook on how to achieve more desirable laser performance by enhancing the stability, damage threshold, and other saturable absorption parameters.

## 2. Passive Modulation Mechanism of SAs

SAs exhibiting an ultrafast nonlinear saturable absorption effect have emerged as the core component for realizing passive QS and ML technologies in lasers. Under the irradiation of an intense laser, the interaction between light and matter no longer follows the Lambert-Beer law but enters the nonlinear region. The relationship between the polarization intensity *P* and the electric field intensity *E* of the material needs to be expanded using a power series:

$$P=\varepsilon_{0}(\chi^{\left(1\right)}E+\chi^{\left(2\right)}E^{2}+\chi^{\left(3\right)}E^{3}+\cdots )$$

Saturable absorption falls under the category of third-order nonlinear optical effects, $\chi^{\left(3\right)}$, which directly relates to the variation in the imaginary part of the material’s complex refractive index (i.e., the absorption coefficient) with light intensity. Its constitutive relationship can be expressed as:

$$\alpha \left(I\right)=\frac{\alpha_{0}}{1+I/I_{S}}$$

where *α*_0_ is the linear absorption coefficient, *I* is the incident laser intensity, *I_s_* is the saturation intensity. The value of the saturation intensity depends on the type of SAs. Upon the incidence of a weak laser pulse on the SA, it demonstrates linear optical absorption. The incident laser wavelength is chosen such that the photon energy exceeds the band-gap energy. As the laser intensity increases, the ground-state electrons are gradually depleted and the excited-state sub-bands become filled. At this point, electrons in the ground state of the materials undergo a transition to an excited state through the absorption of photon energy. Subsequently, certain hot electrons with diminished energy occupy lower energy levels and relax back to the ground state in accordance with the Fermi-Dirac distribution. Given that the transition rate of ground-state electrons is significantly higher than the relaxation rate of excited-state electrons, overall, the quantity of ground-state electrons gradually diminishes, while the quantity of excited-state electrons increases. According to the Pauli exclusion principle, the material can no longer absorb incident photons when the ground-state electrons are depleted and the excited state sub-bands are filled. Consequently, SAs exhibit absorption saturation (i.e., they are “transparent” to light) under high-intensity laser irradiation.

### 2.1. Passive Q-Switching

Building on active Q-switching (QS) with electro-optic Kerr cells [37], P. P. Sorokin et al. used metal phthalocyanine as a SA to achieve passive QS for the first time in a ruby laser in 1964 [19]. Such passive QS technology, which has the advantages of a simple structure, no need for external driving, cost-effectiveness, and durability, rapidly became a key research direction in the field of military and research QS lasers. The generation of QS pulses is dependent on the sudden alteration in the quality factor (*Q*) of the laser resonator:

$$Q=2\pi v_{0}\frac{E}{c\delta E/nL}=\frac{2\pi nL}{\delta \lambda_{0}}$$

where *E* represents the intracavitary energy, *δ* is the optical loss per round trip, *L* is the cavity length, *n* is the refractive index of the intracavity medium, *c* is the speed of light, *λ*_0_ is the central wavelength of lasers in vacuum and *ν*_0_ is the central frequency of lasers. Owing to the high cavity loss (i.e., the low Q-value) during the initial phase of laser pumping, the intracavity gain fails to offset the laser threshold, resulting in the continuous accumulation of the population inversion density at the upper laser level. When intracavitary energy reaches a certain value, SAs become transparent to light and the resonator’s Q-value suddenly increases. Meanwhile, the upper-level population undergoes an avalanche-like transition through stimulated emission, releasing energy instantaneously and producing large-energy QS pulses with durations ranging from a few nanoseconds to multiple microseconds.

### 2.2. Passive Mode-Locking

Passive mode-locking (ML) represents the most prevalently employed technique for attaining ultrashort pulses on the picosecond and even femtosecond scales. Distinct from passive QS [16,17,18], ML attains a fixed phase relationship among all cavity longitudinal modes through multiple rounds of linear amplification of the gain medium and saturable absorption of the SA. The SA acts as a loss modulator that is dependent on light intensity: it exhibits high loss for weak light (such as noise floor or pulse edges), while it saturates and “bleaches” to low loss for strong short pulses. After multiple cavity roundtrips, strong pulses are preferentially amplified, while weak signals are suppressed. This self-amplitude modulation effect, combined with the fast recovery characteristics of the saturable absorber, can continuously compress the pulse front and tail, ultimately forming a stable ultrashort pulse sequence. Simultaneously, the saturable absorber possesses both fast and slow recovery components: the slow recovery component aids in the self-starting of mode-locking, while the fast recovery component is responsible for pulse shaping on the sub-picosecond scale, thereby achieving spontaneous establishment of stable mode-locking from noise and maintaining ultrashort pulse output in a steady state [38].

In 1992, the emergence of SESAMs spurred the rapid development of passive ML technology. Keller et al. utilized molecular beam epitaxy to grow a GaAs layer with saturable absorption properties on a Bragg reflector and incorporated it into a titanium-sapphire laser to obtain ML pulses with a pulse width of 2 ps [22]. In comparison with earlier organic dyes and color-center crystals [19,21], SESAMs are characterized by a simple and compact structure, long lifetime, ultrafast response, as well as self-starting and self-sustaining capabilities. Currently, they have realized large-scale commercial production and are extensively employed in various passively ML lasers [23,24,25]. Nevertheless, it is an undeniable fact that SESAMs are confronted with the issues of fixed operating wavelengths and intricate fabrication processes, which restricts their advancement in the domains of mid-infrared lasers and tunable lasers.

## 3. Research Progress on LDM SAs

**Figure 1:**
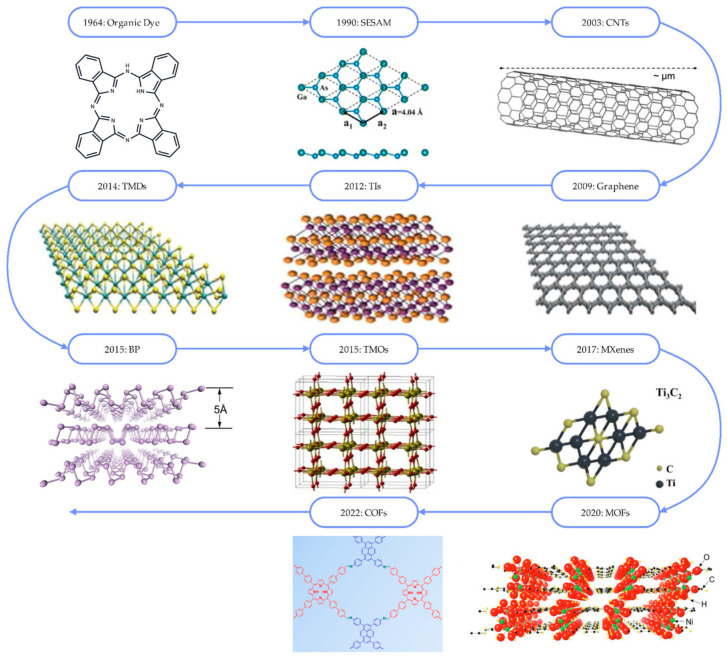
Historical evolution of SA technologies in a chronological order. SESAM (GaAs) [41], CNTs [42], Graphene [40], TIs (Bi_2_Se_3_) [43], TMDs (MoS_2_) [40], BP [44], TMOs (WO_3_) [45], MXenes (Ti_3_C_2_) [46], MOFs [35], COFs [36].

In 2004, the successful application of one-dimensional CNTs-as saturable absorbers in fiber ML lasers drew the attention of researchers to nonlinear nanomaterials [39]. In recent years, LDMs, such as CNTs, graphene, TIs, TMDs, BP, TMOs, MXenes, MOFs and COFs as depicted in Figure 1, have emerged as research focuses for high-performance SAs in laser applications. This can be ascribed to their unique band structures, strong light-matter interactions, and ultrafast carrier dynamics [40]. The subsequent sections focus on reviewing the LDM SAs (especially one-dimensional and two-dimensional) that are of interest, along with their diverse material systems.

### 3.1. Graphene

**Figure 2:**
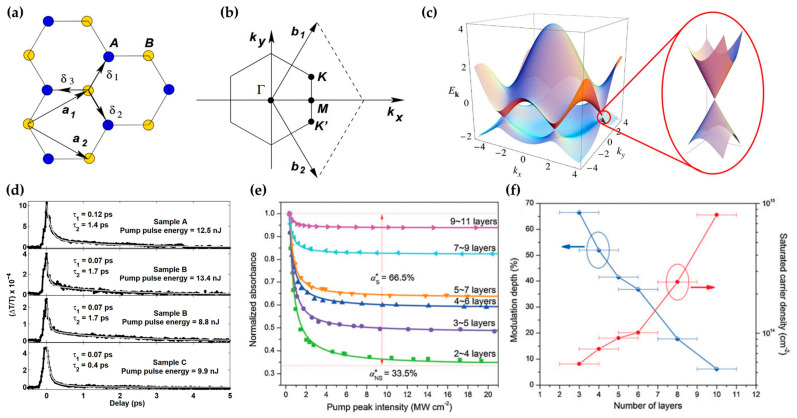
(**a**) The lattice structure of graphene is composed of two interlaced hexagonal lattices [49]. A and B represent two asymmetric carbon atoms, *δ*_1_ *δ*_2_ and *δ*_3_ are the wave vectors of neighboring carbon atoms, and *a*_1_, *a*_2_ are the lattice unit vectors of graphene; (**b**) Corresponding Brillouin zone [49]. The Dirac cones are located at the K and K’ points. (**c**) Energy spectrum for finite values of *t* and *t*’, with *t* = 2.7 eV and *t*’ = 0.2 *t* [49]. Zoom–in of the energy bands close to one of the Dirac points; (**d**) Measured transmittivity transients for samples with different pump power, the initial fast relaxation time *τ*_1_ corresponds to carrier–carrier intraband scattering rates and the slow relaxation time *τ*_2_ correlates with electron–hole interband recombination [48]. (**e**) Nonlinear absorption of graphene films with different number of layers, α*_S_ and α*_NS_ are the saturable and nonsaturable absorption components [26]. (**f**) Modulation depth and saturated carrier density versus number of graphene layers [26].

Graphene is a two-dimensional (2D) honeycomb lattice material composed of a single layer of carbon atoms arranged via sp^2^ hybridization [39,40], as demonstrated in Figure 2a. It is found to be a zero-bandgap semimetal with a linear overlap between valence and conduction bands at the K and K’ points (Dirac points) of the Brillouin zone (refer to Figure 2b,c) [40]. Thus, it exhibits a broadband photoresponse that spans almost the entire optical spectrum [47]. As depicted in Figure 2d, the ultrafast dynamics of photo-excited carriers are further verified, characterized by an initial rapid carrier-carrier intraband scattering time of 70 fs and an electron-hole interband recombination time of 1.7 ps [48]. This indicates that graphene has the potential for the generation of femtosecond ultrafast laser pulses.

**Figure 3:**
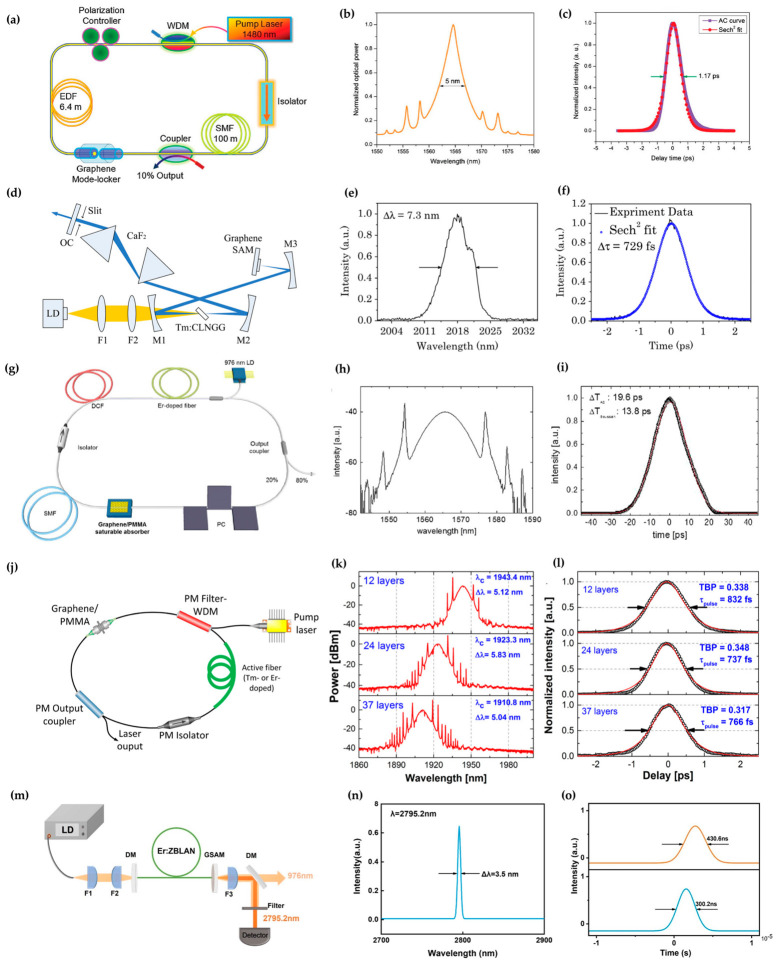
The performance of passively modulated laser based on graphene. (**a**) Schematic of Er^3+^–doped fiber laser based on graphene–SA. (**b**) Spectrum. (**c**) Autocorrelation trace [26]. (**d**) Experimental setup of the mode–locked laser based on the graphene SAM. (**e**) Optical spectrum. (**f**) Autocorrelation trace of the mode–locked pulses [51]. (**g**) Experimental setup of the dissipative soliton Er–doped fiber laser using a graphene/PMMA saturable absorber. (**h**) Optical spectrum. (**i**) Intensity autocorrelation function [59]. (**j**) General schematic of the all–PM fiber laser used in the experiments. (**k**) Measured optical spectra of the TDFL for different number of graphene layers in the saturable absorber. (**l**) Pulse durations [60]. (**m**) Schematic setup of the passively Q–switched Er:ZBLAN fiber laser based on the GSAM. (**n**) Optical spectrum. (**o**) Pulse trains for the 0.3 W and 0.8 W pump power [63].

In 2009, Bao et al. prepared atomic-layer graphene SA by chemical vapor deposition (CVD) and investigated its nonlinear saturable absorption properties (refer to Figure 2e,f) for the first time [26]. It exhibits a relatively larger modulation depth, accompanied by a saturation intensity that is one order of magnitude lower than that of SESAM. These intrinsic merits arising from its 2D structure are more favorable for the generation of ultrashort pulses. Upon integration into an erbium-doped fiber (EDF) laser, the duration of the acquired passive ML pulse is 756 fs, and the central wavelength is 1565 nm with a spectral linewidth of 5 nm (refer to Figure 3a–c). This study verified the application merits of graphene (e.g., broadband modulation, ultrafast relaxation times, excellent nonlinear absorption, low insertion loss and saturation intensity) in ultrafast photonics applications, thereby initiating research on graphene-based passive ML lasers.

Subsequently, the modulation wavelength of graphene-based SAs has undergone a rapid expansion into the near/mid-infrared region. In 2010, the mode-locking of a ceramic Nd:YAG solid-state laser (SSL) at 1064 nm utilizing solution-processed graphene as a SA was demonstrated [50]. In 2012, Ma et al. experimentally demonstrated a passively mode-locking laser with laser pulses as short as 729 fs at 2018 nm based on a graphene SAM [51], as shown in Figure 3d–f. Zhang et al. applied a graphene-polymer composite SA in a Tm-doped fiber (TDF) laser and achieved a passive ML pulse of 3.6 ps operating at a wavelength of 1.94 μm [52]. Later, multiform graphene SAs fabricated through mechanical exfoliation [53], ultrasonic liquid-phase exfoliation [54], and CVD [55] were utilized in ML fiber lasers operating at 2 µm, and the narrowest pulse width attained was 370 fs [53]. In 2013, a monolayer graphene SA was used in a passive ML solid-state Cr:ZnSe laser operating at ~2.5 um [56]. At the same time, a compact, graphene QS Er^3+^-doped ZBLAN fiber laser at 2.78 um was demonstrated [57]. These findings suggest that graphene holds the potential to function as a mid-infrared SA, which combines simplicity, cost-efficiency, and broadband modulation, thus promoting the development of mid-infrared ML lasers.

However, thin film nanomaterials are limited for high-power laser applications due to their low damage threshold and poor environmental stability. In 2013, Tang et al. devised a double-piece single-layer graphene plate and employed a collimating lens to augment the laser beam size on the SA, thereby significantly enhancing the damage threshold of graphene. The maximum output power of 5.2 W stable Q-switching 2 μm TDF laser is realized with pulse energy of up to 18 μJ [58]. Almost at the same time, an evanescent field-coupled graphene/PMMA SA was proposed and delivered ML pulses with a pulse energy of more than 10 nJ at 1565 nm [59], as shown in Figure 3g–i. This scheme mitigates optically induced thermal damage resulting from direct interaction and ensures a long nonlinear interaction length to enhance the pulsating capability of SA.

Since then, graphene-based passive ML lasers have been consistently dedicated to comprehensive breakthroughs in the directions of ultra-short pulse, high pulsed energy, mid-infrared modulation, and multi-functionality. Research studies of Sobon et al. in 2015 showed that the nonlinear optical parameters of SA can be controlled by scaling the number of graphene layers [60]. As shown in Figure 3j–l, 24-layer and 37-layer graphene were used in EDF and TDF lasers to achieve pulses with durations of 737 fs and 345 fs, respectively. The multilayer graphene-based SA exhibits a relatively higher modulation depth and a higher damage threshold than the single-layer one. The shorter pulses of 88 fs are generated directly from a ML EDF laser centered at 1545 nm based on a 60-layer graphene/PMMA SA (with a damage threshold of 460 µJ/cm^2^ and a modulation depth of 11%) [61]. To date, the narrowest pulse of 29 fs has been obtained with an average power of 52 mW, which verifies that graphene serves as a simple, low-cost, and robust SA for applications demanding high temporal resolution [62].

In 2016, Sobon et al. first demonstrated noise-like pulse generation with an intrinsic spectral bandwidth exceeding 60 nm from a graphene SA-driven TDF laser, expanding the applications of graphene-based lasers in the supercontinuum generation area [64]. The largest single-pulse energy reaches 51.5 nJ with an average power of 1.21 W after amplification in an all-fiber system. Chen et al. used a polymer-free graphene oxide film to achieve high-power passive ML pulses of 6.62 ps with a maximum average output power of 3.89 W [65]. The highest pulse peak power reaches 4.85 kW and the pulse energy is 32.14 nJ.

In the same year, a multilayer graphene was employed in Er^3+^-doped ZBLAN fiber laser to obtain 42 ps pulses at 2.78 µm [66]. In 2018, Pawliszewska et al. used graphene SA in a Ho^3+^-doped fiber laser to achieve stretched-pulse output of 190 fs at 2060 nm by implementing dispersion management [67]. To further broaden the operating wavelength range of graphene, A. V. Pushkin et al. reported a passive ML Fe:ZnSe laser implemented using graphene SA for the first time in 2020. The laser operates at 4.4 µm with a pulse duration of about 732 fs and a repetition frequency of 100 MHz [68].

In 2021, graphene was employed in a synchronized ML fiber laser consisting of both erbium and thulium-doped fiber laser, generating 700 fs ultrashort pulses at 1563.5 nm with 24 nJ pulse energy and 1.77 ps ultrashort pulses at 1931.9 nm with 26 nJ pulse energy. The success of this work provide a better insight in generating multiple ML lasers with different wavelength [69]. In 2022, Jin et al. proposed a low-temperature plasma-enhanced CVD to directly deposit a high-quality, transfer-free graphene SA mirror, achieving stable passive QS in an Er^3+^-doped ZBLAN fiber laser at 2.8 μm with the pulse duration of 300.2 ns (refer to Figure 3m–o) and a slope efficiency of 17.4%. The average output power, pulse energy and peak power were 142 mW, 2.33 μJ and 7.76 W [63]. This approach resolved the key challenges of impurity introduction and poor modulation stability associated with conventional transfer fabrication, providing a novel and feasible approach for developing high-performance mid-infrared pulsed laser devices.

Table 1 summarizes the achievements of passively modulated lasers based on graphene saturable absorbers. To date, graphene-based passively modulated lasers have covered the primary laser wavelength bands ranging from 1 μm to 4.4 μm (see in Table 1), establishing a comprehensive suite of technical solutions that includes soliton mode locked, stretched pulses, noise-like pulses, QS and multi-wavelength synchronous ML, demonstrating significant application value across various fields of scientific research.

### 3.2. Transition Metal Dichalcogenides

**Figure 4:**
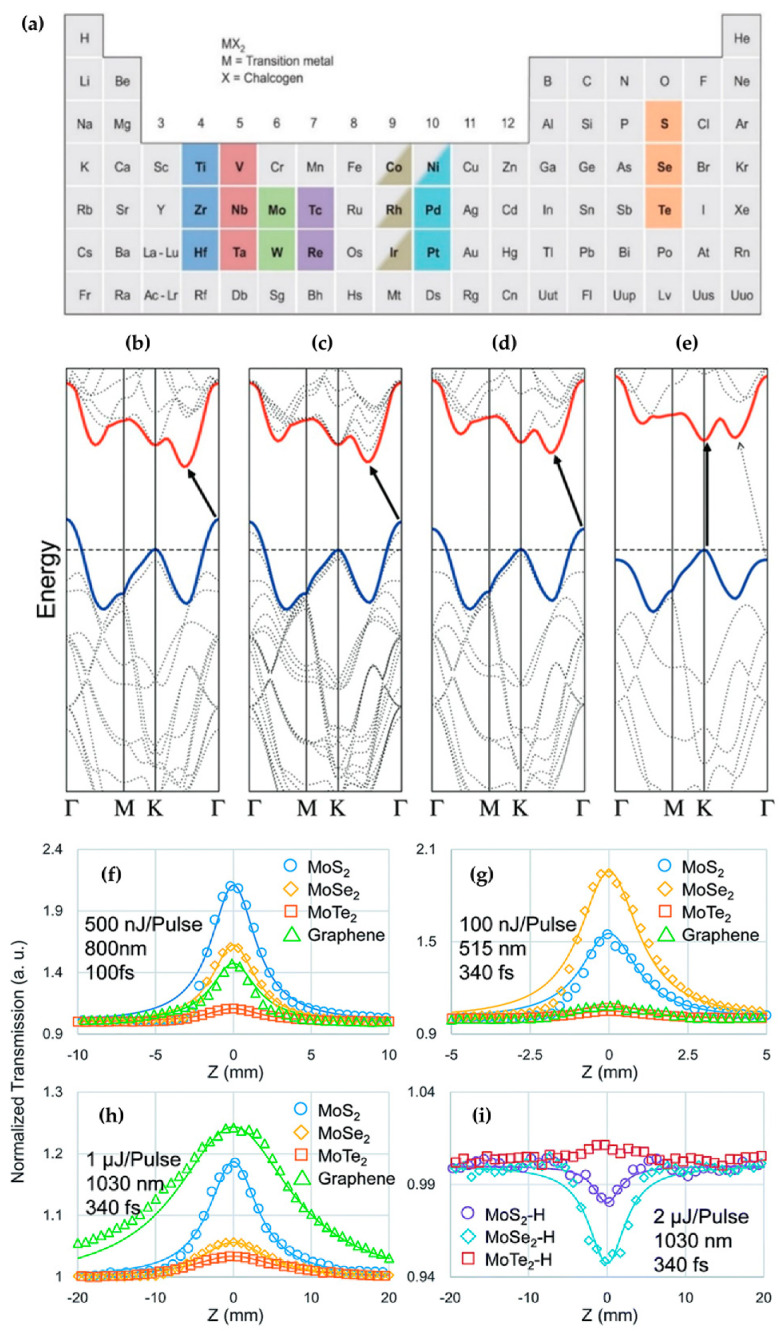
(**a**) The positions of the elements represented by M and X in the periodic table; Calculated band structures of (**b**) bulk MoS_2_, (**c**) quadrilayer MoS_2_, (**d**) bilayer MoS_2_, and (**e**) monolayer MoS_2_ [88]; (**f**–**i**) OA Z–scan results of the MoX_2_ dispersions in fs region. Samples in (**f**–**h**) exhibit obvious SA response, while showing TPA response in (**i**) for the MoS_2_ and MoSe_2_ dispersions with a higher speed centrifugation treatment (10,000 rpm) [89].

TMDs constitute a category of two-dimensional layered compounds with the chemical formula MX_2_, in which M denotes a transition metal element in groups 4–10 (e.g., Mo, W, V, as shown in Figure 4a) and X represents a chalcogen element (e.g., S, Se, Te). The X atoms and M atoms are strongly connected by covalent bonds within the layers, while the bonds between adjacent layers are formed through weak van der Waals force [87]. Unlike gapless graphene, TMDs exhibit layer-dependent tunable bandgaps. Figure 4b–e illustrate the band structures of molybdenum disulfide (MoS_2_) with different numbers of layers [88]. It transitions from an indirect bandgap in bulk form (~1.2 eV) to a direct bandgap (~1.8 eV) in the monolayer. In 2013, Wang et al., measured the saturable absorption of few-layer MoX_2_ (X = S, Se and Te) nanosheets by Z-scan system at 400 nm, 515 nm, 800 nm, and 1030 nm [28,89]. The results are presented in Figure 4f–i. At relatively short visible wavelengths (e.g., 400 nm, 515 nm, 800 nm), few-layer MoX_2_ (X = S, Se) can demonstrate a stronger nonlinear optical response than graphene under specific experimental conditions. However, in the near-infrared of 1030 nm, graphene generally maintains superior or comparable performance owing to its gapless Dirac band structure and ultrafast carrier dynamics.

Research on passively modulated lasers using a TMDs-based SA began in 2014. The key breakthroughs in the initial stage were concentrated on validating the saturable absorption characteristics of classical Group VI binary TMDs, optimizing fabrication techniques and integration strategies, and expanding the multi-band application boundaries. Zhang et al. synthesized few-layered MoS_2_ nanoplatelets by the hydrothermal exfoliation method and deposited them onto the end facet of the optical fiber of a YDF laser at 1054.3 nm for the generation of 800 ps ML pulses [90]. A few-layer MoS_2_ polymer composite SA was prepared and used in a passive QS YDF laser with over 100 nJ pulse energy and 40 nm of tunability, tunable from 1030 to 1070 nm. This highlights the potential of few-layer MoS_2_ as a wideband saturable absorber [91]. A multilayer MoS_2_ SA was fabricated via the CVD method and transferred onto the end-face of a fiber connector within an EDF laser. Leveraging its outstanding saturable absorption property, stable mode-locking with a pulse duration of 1.28 ps and a repetition rate of 8.288 MHz was achieved at a central wavelength of 1568.9 nm [92]. A MoS_2_ sample with over 30 layers (featuring an absolute modulation depth of 27%) was fabricated via the pulsed laser deposition (PLD) technique and employed as SAs in passive QS SSLs at wavelengths of 1.06 μm (Nd:GdVO_4_), 1.42 μm (Nd:YGG), and 2.1 μm (Tm:Ho:YGG) [93]. In addition, several typical integration methods, such as direct deposition onto microfibers [94], side-polished fibers [95], and also deposition onto quartz/BK7 glass [96], have been reported and used to QS and ML both bulk and fiber lasers from 1030 to 2100 nm, enabling laser pulse generation at kHz to GHz repetition rates and with few microsecond to sub-picosecond pulse durations.

In 2015, Chen et al. systematically compared the nonlinear optical parameters and passive QS performance of four representative Group VI binary TMD materials (e.g., MoS_2_, MoSe_2_, WS_2_, and WSe_2_) [97]. The experimental results revealed that MoSe_2_ exhibited the highest modulation depth (6.73%), while WS_2_ demonstrated the best long-term operational stability. This study presented systematic experimental evidence for material selection and performance optimization within the TMD family.

**Figure 5:**
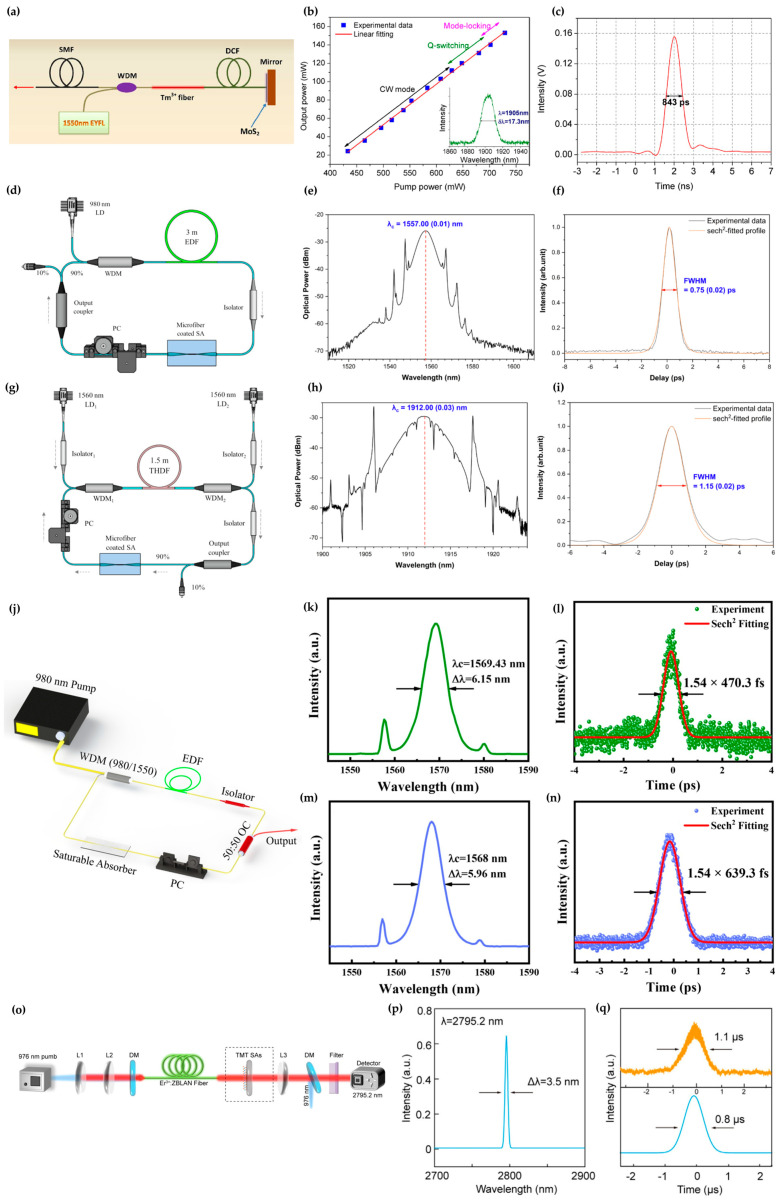
The performance of passively modulated laser based on TMDs. (**a**) Experimental setup of the mode–locked Tm^3+^ fiber laser. (**b**) Output of the mode–locked Tm^3+^ fiber laser. Inset shows the laser spectrum of the mode–locked Tm^3+^ fiber laser. (**c**) Single pulse of the MoS_2_ [98]. (**d**) Configuration of the EDFL cavity. (**e**,**f**) The output characteristics of the mode–locked EDFL. (**g**) Configuration of the THDFL cavity. (**h**,**i**) The output characteristics of the mode–locked THDFL [118]. (**j**) Experimental setup of an Er–doped fiber laser. (**k**) Optical spectrum. (**l**) Autocorrelation trace based on the MoS_2_ SA. (**m**) Optical spectrum. (**n**) Autocorrelation trace based on the MoSe_2_ SA [119]. (**o**) Schematic setup of the passively Q–switched Er^3+^–doped ZBLAN fiber laser incorporating TMT–SAs. (**p**) Optical spectrum. (**q**) Single pulse profiles of TaTe_2_–SA and ZrTe_3_–SA [112].

In the same year, benefiting from their unique electronic structures, strong light–matter interactions, and excellent nonlinear optical absorption properties, TMDs became a research focus in the field of mid-infrared SAs. Tian et al. first integrated a multilayer MoS_2_ SA into a 2 μm TDF laser and successfully realized stable ML pulses at a center wavelength of 1905 nm, with a pulse width of 843 ps and a signal-to-noise ratio greater than 55 dB [98]. The cavity schematic, output spectrum and autocorrelation trace are shown in Figure 5a–c. Jung et al. demonstrate the use of an all-fiberized, thulium–holmium co-doped fiber mode-locked laser based on a WS_2_-deposited side-polished fiber. The stable ML pulses with a temporal width of 1.3 ps at a repetition rate of 34.8 MHz were obtained at 1941 nm [99]. Subsequently, Fan et al. realized a passive QS operation in an Er:Lu_2_O_3_ laser at 2.84 μm by employing a MoS_2_ SA. Under the laser pump power of 7.61 W, an average output power of 1.03 W was generated with a pulse duration of 335 ns and a repetition rate of 121 kHz, resulting in a pulse energy of 8.5 μJ [100]. These works provided the experimental confirmation of the broadband modulation capability of TMD materials in the mid-infrared spectral range, laying a core experimental foundation for the application of TMDs in ultrafast mid-infrared lasers.

In the subsequent two years, the device structures based on TMDs for passive ML lasers were further optimized. In 2017, WS_2_ was deposited on the surface of a tapered fiber to form the evanescent field using the pulsed laser deposition (PLD) method [101]. A fiber-taper WS_2_ SA with a large modulation depth of 35.1% was fabricated to support ultrashort pulse generation. The shortest pulse duration of 67 fs was obtained, with a 114 nm spectral width and a 93 dB signal-to-noise ratio (SNR). This indicates that the fiber-taper WS_2_ SA with a large modulation depth is a promising photonic device for ML fiber lasers with a wide spectrum and ultrashort pulse duration. WSe_2_ films that are both large-scale and high quality are prepared by CVD method and tightly transferred onto the side wall of a microfiber to form a hybrid structure. The integrated microfiber-WSe_2_ device realized strong evanescent wave interaction and showed a large modulation depth of 54.5%. Stable soliton ML pulses are generated and the pulse durations of 477 fs (at 1.5 μm) and 1.18 ps (at 2.0 μm) are demonstrated, which suggests that the large-area and highly crystalline WSe_2_ films afford an excellent broadband SA for ultrafast photonic applications [102]. At the same time, a type of microfiber-based WTe_2_ SA fabricated by the magnetron-sputtering deposition (MSD) method was reported, which had a large modulation depth of 34.3%. High-performance SAs with large modulation depths are beneficial for high-energy wave-breaking free pulse generation. Consequently, pulses were generated with a pulse duration, pulse energy, and average output power of 229 fs, 2.14 nJ, and 57 mW in the 1.5 μm regime, and 1.3 ps, 13.8 nJ, and 212 mW in the 2 μm regime, respectively [103,104]. In 2019, Lv et al. reported a novel type of MoS_2_-doped sol–gel glass SA with a high optical damage threshold, which was fabricated using the sol–gel technique. Stable mode-locked pulse trains were successfully generated in the normal dispersion regime, with a pulse width of 13.8 ps and an average output power of 34.6 mW. It potentially provides a new approach to improving the optical damage threshold and long-term working stability of broadband SAs [105].

The studies since 2019 were not merely restricted to classical Group VI binary TMDs but rather involved a comprehensive expansion of the Group 4–10 TMDs material system (e.g., ZrSe_2_ [106], ZrTe_2_ [107], HfS_2_ [108], HfSe_2_ [109], VSe_2_ [110], VTe_2_ [111], TaTe_2_ [112], ReSe_2_ [113], PdSe_2_ [114], PdTe_2_ [115]) and ternary TMD alloy systems (e.g., MoWSe_2_ [116,117]). The emphasis lies on the advancements in ultrashort pulse width, ultra-high repetition rate, and high signal-to-noise ratio laser output. Simultaneously, it entails accomplishing the customization of materials and devices for specific application scenarios to propel TMD-based lasers from laboratory proof-of-concept demonstrations to practical applications. Table 2 summarizes the achievements of passively modulated lasers based on TMDs SAs.

In 2019, a MoWSe_2_ ternary TMD alloy SA was developed, and its broadband modulation capability across the near-infrared to mid-infrared spectral range was first verified [116]. The continuous adjustment of the band structure and nonlinear optical parameters via bimetallic alloying paves a new technological path for the performance optimization of TMDs. In 2022, Ahmad et al. used a HfSe_2_ SA through evanescent-field coupling to obtain an ultrashort ML pulse of only 0.75 ps at 1.5 μm and of 1.15 ps at 2 μm, with signal-to-noise ratios exceeding 60 dB in both wavelength bands [118]. The cavity schematic, output spectrum and autocorrelation trace are shown in Figure 5d–i. This work confirmed that Group IV TMDs possess higher carrier mobilities and superior nonlinear modulation performance compared with traditional Group VI materials, substantially broadening the material research boundaries of TMDs.

In 2024, Chen et al. systematically compared the ML laser performance of MoS_2_ and MoSe_2_ [119]. As shown in Figure 5j–n, MoS_2_ exhibits a relatively shorter carrier recovery time (164.6 ps), thereby enabling the generation of narrower ultrashort pulses with a width of merely 470 fs. This characteristic renders it more conducive to applications in ultrashort pulse lasers. MoSe_2_ demonstrates lower non-saturable loss and attains higher laser slope efficiency, rendering it more suitable for high-power, high-efficiency laser applications. This study offers accurate experimental and theoretical guidance for the selection of TMDs in diverse application scenarios.

In 2025, Wang et al. demonstrated stable operation from fundamental ML to up to 86th-order harmonic ML in an EDF laser based on a MoWSe_2_ ternary TMD SA [120]. An ultrahigh repetition-rate pulse reaching up to 374.8 MHz was successfully attained, which demonstrated the substantial application potential of TMDs in high-repetition-rate ultrafast lasers, high-speed optical communications, and ultra-precision optical metrology.

In the same year, Liu et al. put forward a preparation procedure for Atomic Oxygen- Passivated 2D Zr/Ta Telluride crystal SAM intended for high-power mid-infrared lasers. This methodology entails the direct in situ growth of large-area, high-quality 2D ZrTe_3_ and TaTe_2_ films on CaF_2_ substrates via CVD technology. This method circumvents wet-coating procedures in various previously reported SAs, thereby alleviating the performance degradation induced by impurities. After oxygen plasma treatment, a dense and stable oxide protective layer was formed on the material surface, significantly enhancing the nonlinear optical performance and environmental robustness of SA [112]. The schematic diagram of the cavity, the output spectrum, and the pulse envelope are presented in Figure 5o–q. This study provides a new technical pathway for developing highly stable 2D material SAs for high-power mid-infrared pulsed lasers. Table 2 summarizes the achievements of passively modulated lasers based on TMDs SAs.

**Table 2:** Performance summary of passively modulated lasers based on TMDs absorbers. λ_0_ is the center wavelength, τ_p_ is the pulse width, f_rep_ is the repetition frequency, E_p_ is the pulse energy, P_ave_ is the average output power.

SA	Gain Medium	λ_0_ (nm)	τ_p_ (ps)	f_rep_ (KHz)	E_p_ (nJ)	P_ave_ (mW)	Thickness	References
MoS_2_	Yb^3+^ silica fiber	1029.8	13.8	22,440	-	34.6	few-layer	[105]
MoS_2_	Yb^3+^ silica fiber	1042.6	656	6740	-	25	0.6~2.1 nm	[94]
MoS_2_	Nd^3+^: GdVO4	1060	9.7 × 10^5^	732	310	227	few-layer	[93]
	Nd^3+^: YGG	1420	7.29 × 10^5^	77	670	52		
	Tm^3+^: Ho^3+^: YGG	2100	4.1 × 10^5^	149	1380	206		
MoS_2_	Yb^3+^ silica fiber	1070	2.88 × 10^6^	74	100	9.36	2~4 nm	[91]
MoS_2_	Er^3+^ silica fiber	1568.9	1.28	8288	-	-	3.42 nm	[92]
MoS_2_	Tm^3+^ silica fiber	1905	843	9670	15.5	150	4 layers	[98]
MoS_2_	Tm^3+^ silica fiber	1927	1.51	13,900	-	5	1.86 nm	[121]
MoS_2_	Tm^3+^/Ho^3+^ silica fiber	1979	1.97	9120	2.2	20	5~6 layers	[122]
MoS_2_	Er^3+^: ZBLAN	2754	8.06 × 10^5^	70	2000	140	4 layers	[123]
MoS_2_	Er^3+^: Lu_2_O_3_	2840	3.35 × 10^5^	121	8500	1030	4~10 layers	[100]
MoS_2_	Er^3+^ silica fiber	1560	1.35 × 10^7^	41.452	184.7	0.77	1~5 layers	[97]
MoSe_2_		1559	6.5 × 10^6^	66.847	369.5	2.45		
WS_2_		1562	6.7 × 10^6^	77.925	1179.4	6.41		
WSe_2_		1561	9.18 × 10^6^	85.365	484.8	3.16		
MoS_2_	Er^3+^ silica fiber	1569.4	0.47	22,380	-	5.63	31 nm	[119]
MoSe_2_		1568	0.639	22,310	-	7.1	25 nm	
MoSe_2_	Nd^3+^: GdVO_4_	1340	4.2 × 10^5^	238	227	52.6	10 layers	[124]
MoSe_2_	Tm^3+^/Ho^3+^ silica fiber	1912	0.92	18,210	-	-	52 nm	[125]
MoSe_2_	Tm^3+^ silica fiber	1945.35	0.98	23,530	0.39	8.2	-	[126]
MoSe_2_	Tm^3+^/Ho^3+^ silica fiber	1982	4300/3800	2200	2.31	5.1	7 nm	[127]
VSe_2_	Tm^3+^/Ho^3+^ silica fiber	1912	1.4	11,600	-	0.8	-	[110]
WTe_2_	Tm^3+^/Ho^3+^ silica fiber	1909.8	1.77	11,540	0.304	3.51	6 layers	[128]
WTe_2_	Tm^3+^ silica fiber	1915.5	1.25	18,720	2.13	39.9	290 nm	[103]
WTe_2_	Tm^3+^ silica fiber	1951.5	1.31	13,070	8.26	108.1	166 nm	[129]
WTe_2_	Er^3+^ silica fiber	1560.5	1.77 × 10^6^	55.56	18.09	1.01	213.6 nm	[130]
WS_2_	Er^3+^ silica fiber	1540	0.067	135,000	-	-	-	[101]
WS_2_	Tm^3+^/Ho^3+^ silica fiber	1910	0.825	15,490	0.0182	2.82	6 nm	[131]
WS_2_	Tm^3+^/Ho^3+^ silica fiber	1925	1.3	34,800	0.017	0.6	6 nm	[99]
WS_2_	Tm^3+^: LuAG	2012.9	6.6 × 10^5^	63	17,000	1080	4 nm	[132]
WS_2_	Er^3+^: Y_2_O_3_	2716.3	7.2 × 10^5^	29.4	7920	233.5	5.52 nm	[7]
WSe_2_	Er^3+^ silica fiber	1552.6	0.39	24,500	0.042	1.03	0.8 nm	[133]
WSe_2_	Er^3+^ silica fiber	1555	0.7	23,950	0.21	5.28	-	[134]
WSe_2_	Er^3+^ silica fiber	1556.4	0.477	14,020	-	-	1~3 layers	[102]
	Tm^3+^ silica fiber	1886.2	1.18	11,,360	-	32.5		
WSe_2_	Er^3+^: CaF_2_	2756.8	8.4 × 10^5^	46	2750	104	0.73 nm	[135]
ReSe_2_	Er^3+^ silica fiber	1559.8	0.992	28,179	0.02	0.58	-	[113]
MoWSe_2_	Er^3+^ silica fiber	1531.8	-	4360	0.109	0.47	1.8 nm	[120]
MoWSe_2_	Er^3+^ silica fiber	1554	1.9 × 10^6^	48	11.8	1.1	few-layer	[116]
	Tm^3+^ silica fiber	1964	2.4 × 10^6^	61.5	85.3	5.2		
TiS_2_	Er^3+^ silica fiber	1569.5	1.04	5340	5.05	-	9 nm	[136]
PtS_2_	Er^3+^ silica fiber	1530	1.17	5120	0.368	1.86	-	[137]
PtSe_2_	Er^3+^: ZrF_4_	2783.2	1.04 × 10^6^	93.1	10,020	932.7	61.9~101.2 nm	[138]
NiS_2_	Er^3+^ silica fiber	1560.2	0.524	21,100	1.86	39.2	3~6 layers	[139]
NiTe_2_	Er^3+^ silica fiber	1567.3	1.07	22,450	0.176	3.96	5 nm	[140]
	Tm^3+^ silica fiber	1966	3.38	20,660	0.415	8.59		
NbS_2_	Er^3+^ silica fiber	1565.5	0.753	22,730	-	1.45	10 nm	[141]
	Tm^3+^ silica fiber	1961.4	5.77	20,230	0.275	5.56		
HfS_2_	Tm^3+^/Ho^3+^ silica fiber	1916.3	1.509	11,100	0.05	0.56	few-layer	[108]
HfSe_2_	Er^3+^ silica fiber	1557	0.75	14,800	0.139	2.1	few-layer	[118]
	Tm^3+^/Ho^3+^ silica fiber	1912	1.15	12,500	0.108	1.36		
HfSe_2_	Tm^3+^ silica fiber	1963.4	1.53	8910	0.252	2.25	65 nm	[109]
VTe_2_	Tm^3+^: YLF	1907.1	5.63 × 10^5^	62.5	3790	237	25 nm	[111]
	Er^3+^: ZBLAN	2795.9	7.49 × 10^5^	129.8	3100	402.8		
MoTe_2_	Er^3+^ silica fiber	1559.5	0.229	26,601	2.14	57	11.6 nm	[104]
	Tm^3+^ silica fiber	1934.8	1.3	15,370	13.8	212		
MoTe_2_	Tm^3+^ silica fiber	1930	0.952	14,353	2.56	36.7	6.8 nm	[142]
1T′-MoTe_2_	Er^3+^ silica fiber	1595	1.3 × 10^6^	135	-	8.45	6.1 nm	[143]
2H-MoTe_2_			0.537	32,800	-	2.5		
Mo_0.5_W_0.5_S_2_	Tm^3+^: YAP	1936	6.33 × 10^5^	146.82	21,800	3255	5.3 nm	[117]
PdTe_2_	Er^3+^ silica fiber	1559.6	0.57	12,070	0.043	0.524	13.1 nm	[115]
	Tm^3+^ silica fiber	1896.4	1.59	14,950	0.63	9.39		
PdSe_2_	Er^3+^ silica fiber	1557	1.31	12,560	-	1.8	10 nm	[114]
ZrSe_2_	Er^3+^ silica fiber	1561.8	12.5	21,220	0.54	11.37	few-layer	[106]
ZrTe_2_	Er^3+^ silica fiber	1560.7	37,500	1220	42.6	52	4.8 nm	[107]
TaTe_2_	Er^3+^: ZBLAN	2795	6.8 × 10^5^	188.9	-	550	30 nm	[112]
ZrTe_3_			3.13 × 10^5^	196.2	-	630		

### 3.3. Black Phosphorus

**Figure 6:**
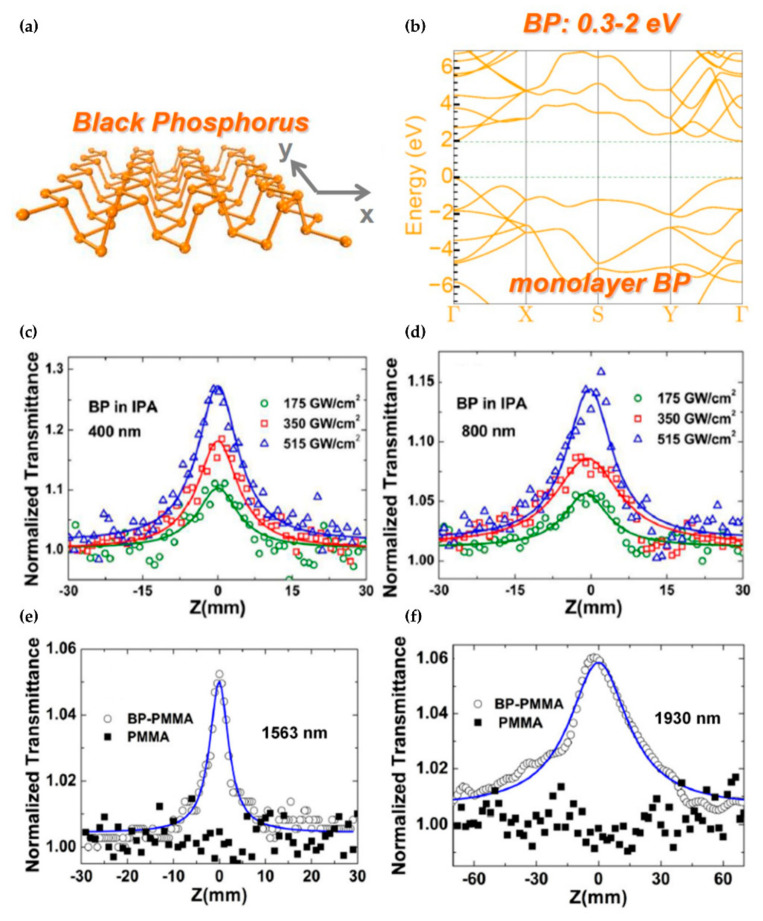
(**a**) Atomic structure of BP. (**b**) Band gap of BP [40]. (**c**,**d**) are the open aperture Z–scan measurements of BP NPs dispersions under different intensities at 400 nm and 800 nm, respectively; (**e**) Relation between normalized transmittance and input intensity for BP NPs dispersions at 800 nm; (**f**) The open aperture Z–scan measurements of BP NPs dispersions in IPA, NMP and EA at intensities of 515 GW/cm^2^ [27].

2D BP is a layered crystalline material with a puckered honeycomb atomic structure, as shown in Figure 6a [40]. In 2014, the first application of few-layer BP with a thickness down to a few nanometers in field-effect transistors garnered substantial attention within the optoelectronic domain [44]. It was found that BP is a high-mobility semiconductor with a direct bandgap sensitivity depending on the number of layers from 0.3 (bulk) to 2.0 eV (single layer), as illustrated in Figure 6b. The narrow bandgap enables it to function as a SA, which can be employed in a wider wavelength operation range of 0.8~4 μm.

In 2015, Zhang et al. utilized the liquid-phase exfoliation technique to reduce the bulk BP to a thickness of roughly ten layers and experimentally verified its broadband and enhanced saturable absorption at wavelengths of 400~1930 nm for the first time [27]. As depicted in Figure 6c–f, the saturation modulation depths were experimentally determined to be 27.6% at 400 nm, 12.4% at 800 nm, 19.5% at 1563 nm, and 16.1% at 1930 nm, respectively. Additionally, the carrier dynamics in BP suspension were studied by pump-probe experiments at a wavelength of 1550 nm, and its ultrafast recovery time was observed (*τs* = 24 ± 2 fs) [144]. Compared with graphene, BP possesses a faster recovery time and a larger modulation depth.

**Figure 7:**
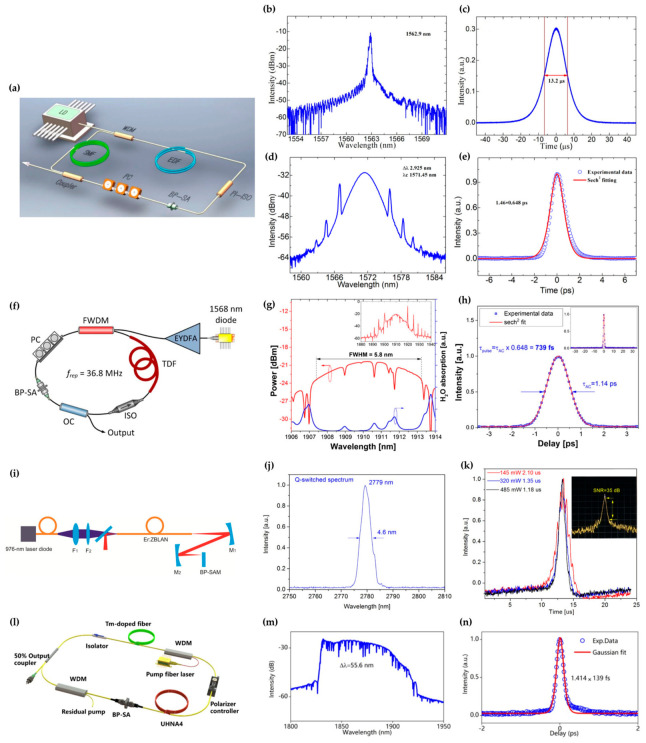
The performance of passively modulated laser based on BP. (**a**) Experimental setup of the BP–SA based Q–switching Er^3+^–doped fiber laser. (**b**) Optical spectrum of QS. (**c**) Single pulse profile of QS. (**d**) Optical spectrum of ML. (**e**) Single pulse profile of ML [145]. (**f**) Experimental setup of the mode–locked Tm^3+^–doped fiber laser. (**g**) Optical spectrum. (**h**) Autocorrelation trace [146]. (**i**) The schematic of the passively Q–switched Er^3+^:ZBLAN fiber laser. (**j**) Optical spectrum. (**k**) Corresponding pulse profiles and the RF spectrum [147]. (**l**) Schematic diagram of the BP–SA based near zero–dispersion fiber laser. (**m**) Optical spectrum. (**n**) Autocorrelation trace [157].

In the same year, the BP SAs prepared by mechanical exfoliation or liquid-phase-exfoliated methods were applied in pulsed lasers operating in the near/mid-infrared spectral region. Chen et al. obtained either the passive QS (with maximum pulse energy of 94.3 nJ) or the passive ML operation (with pulse duration down to 946 fs) in a 1.5 μm EDF laser based on a few-layer BP [145]. The cavity schematic, output spectrum and autocorrelation trace are shown in Figure 7a–e. Sotor et al. applied a BP SA to a 2 μm TDF laser and obtained ML pulses with a width of 739 fs [146] (refer to Figure 7f–h). Qin et al. then used a multilayer BP (with a modulation depth of 15% and saturation fluence of 9 μJ/cm^2^) to achieve a passive QS pulse of 1.18 μs at 2.8 μm in an Er^3+^:ZBLAN fiber laser [147] (refer to Figure 7i–k), which makes it a potential broadband SA for pulsed lasers, especially in the mid-infrared spectral regime.

During this period, researchers also developed novel fabrication methods, such as the evanescent field optical deposition method (e.g., microfiber optical deposition [148] and the BP-polymer composite films method [149]), to achieve a long nonlinear interaction length and high integration encapsulation of BP with fiber devices. This systematically enhances the core nonlinear parameters of BP, including the thermal damage threshold, modulation depth, and saturation fluence. Broadband BP SAMs were successfully fabricated through deposition on a high-reflection or high-transmittance mirror and employed in passively modulated bulk lasers operating at 639 nm [150], 1 μm [151,152,153,154], 2 μm [150,155], and 2.4 μm [156]. This offers a potential approach for realizing ultrafast solid-state lasers within the visible to mid-infrared wavelength range.

Since 2016, research on SAs has focused on two fundamental directions: rectifying the inherent deficiencies of BP and improving its laser-modulated performance (e.g., ultra-short pulse, high peak power, expansion of the mid-infrared wavelength range, and multi-functionality). Su et al. demonstrated a BP-based mode-locked solid-state laser with pulses as short as 272 fs at a central wavelength of 1053.4 nm. The peak power of 23.8 MW is the highest among BP-based mode-locked lasers so far [153]. In 2018, Qin et al. demonstrated a mid-infrared BP SAM by transferring liquid-phase exfoliated BP flakes onto a gold-coated mirror and realized QS and ML operation in an Er:ZBLAN laser at 3.5 μm wavelength for the first time. The results of this research showed that BP has great potential as a mid-infrared SA beyond 3 μm wavelength [158].

Jin et al. achieved the shortest pulse duration of 102 fs by using a BP SA fabricated with a highly functional inkjet printing technology in an EDF laser ring cavity at 1555 nm [159]. Although the ultrafast laser utilizing BP as a SA has undergone substantial development due to its broadband absorption characteristics and ability to shorten pulse width, its vulnerability to environmental factors, such as humidity and temperature, has been gradually revealed through increasing research, thereby further restricting its application in high-power scenarios. In 2020, Zhang et al. encapsulated inkjet-printed BP films with a parylene-C passivation layer to achieve long-term environmental stability [157]. Through intracavity dispersion management, they obtained mode-locked pulses as short as 139 fs in a 2 μm TDF laser, fully demonstrating the key advantage of BP in ultrashort pulse generation. The cavity schematic, output spectrum, and autocorrelation trace are shown in Figure 7l–n. Furthermore, a variety of rich pulsed dynamical behaviors, including switchable dual-wavelength and harmonic mode-locking, have been sequentially demonstrated in BP-based lasers [160,161], facilitating their advancement towards multi-scenario practical applications. Table 3 summarizes the achievements of passively modulated lasers based on BP SAs. Overall, BP-based SAs have provided a new technical approach for the development of infrared ultrafast pulsed lasers due to their unique optoelectronic advantages. However, resolving their intrinsic defects through material encapsulation and modification, fabrication process optimization, and cavity design innovation remains the core research direction in this field for the future.

**Table 3:** Performance summary of passively modulated lasers based on BP absorbers. λ_0_ is the center wavelength, τ_p_ is the pulse width, f_rep_ is the repetition frequency, E_p_ is the pulse energy, P_ave_ is the average output power.

SA	Gain Medium	λ_0_ (nm)	τ_p_ (ps)	f_rep_ (KHz)	E_p_ (nJ)	P_ave_ (mW)	Thickness	References
BP	Yb^3+^: CYA	1046	6.2 × 10^5^	113.6	-	37	3~9 nm	[151]
BP	Yb^3+^: Lu^3+^: CALGO	1053.4	0.272	23,800	6.48	23.8	5 nm	[153]
BP	Nd^3+^: YVO_4_	1064.1	6.1	140,000	3.29	460	5 nm	[154]
BP	Nd^3+^: GdVO4	1342	9.42	58,140	3	350	5 nm	[162]
BP	Er^3+^ silica fiber	1532~1570	0.94	4960	-	5.6	0.6~2 nm	[148]
BP	Er^3+^ silica fiber	1542.4	1 × 10^7^	18.5	90	1.4	15~20 nm	[163]
		1543.2	1.2 × 10^7^	9.4	135	1.35		
BP	Er^3+^ silica fiber	1555	0.102	23,900	0.071	1.7	3.37	[159]
BP	Er^3+^ silica fiber	1558	2.18	15,590	-	0.077	10 nm	[164]
BP	Er^3+^ silica fiber	1559.5	0.67	8770	-	53	2.8~3.8 nm	[165]
BP	Er^3+^ silica fiber	1560.7	0.57	6880	0.74	5.1	200 nm	[166]
BP	Er^3+^ silica fiber	1561	2.66	1000	7.35	7.38	4~5 nm	[167]
BP	Er^3+^ silica fiber	1561~1564	2.98 × 10^6^	30.098	283.91	8.545	few-layer	[168]
BP	Er^3+^ silica fiber	1563	1.32 × 10^7^	10.42	58	0.6	25 layers	[145]
		1571	0.946	5960	-	-	15 layers	
BP	Zr^4+^/Er^3+^ silica fiber	1602	3.46	1000	9.89	9.89	few-layer	[169]
BP	Er^3+^: YAG	1645	2.8 × 10^6^	34	10,000	330	4~7 nm	[170]
BP	Tm^3+^ silica fiber	1859	0.139	20,950	0.97	20.4	3.4 nm	[157]
BP	Tm^3+^/Ho^3+^ silica fiber	1898	1.58	19,200	-	2.25	50 nm	[171]
BP	Tm^3+^ silica fiber	1910	0.739	36,800	0.04	1.5	300 nm	[146]
BP	Tm^3+^ silica fiber	1941	4.38 × 10^6^	27.82	500	14	8.88 nm	[172]
BP	Tm^3+^ silica fiber	1948	5.6 × 10^6^	28.1	130	3.8	-	[173]
BP	Tm^3+^: YAP	1969/1979	1.81 × 10^5^	81	39,500	3100	10 nm	[160]
BP	Tm^3+^ silica fiber	2009	3.12 × 10^6^	11.6	3320	38.5	-	[155]
BP	Ho^3+^ silica fiber	2094	1.3	29,100	0.379	11	16~25 nm	[161]
BP	Cr^2+^: ZnSe	2400	1.89 × 10^5^	176	205	36	6~8 nm	[156]
BP	Er^3+^: ZBLAN	2779	1180	63	7600	485	5~20 nm	[147]
BP	Er^3+^: SrF_2_	2790	7.02 × 10^5^	77.03	2340	180	3~9 nm	[174]
BP	Er^3+^: CaF_2_	2793.8	9.548 × 10^5^	41.93	4250	-	3~9 nm	[175]

### 3.4. Other Low-Dimensional Materials

Beyond graphene, TMDs, and BP, several other LDMs have been widely applied in passively modulated pulsed lasers, including CNTs, TIs, TMOs, and MXenes. Table 4 summarizes the achievements of passive ML lasers based on other LDM SAs.

**Figure 8:**
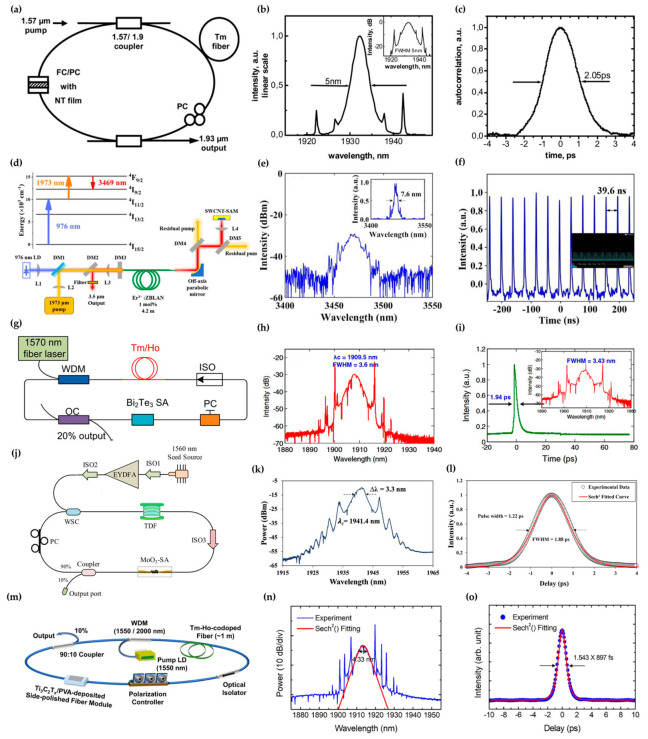
The performance of passively modulated laser based on CNTs, Tis, TMOs and MXenes. (**a**) Scheme of the ring–cavity Tm^3+^–doped fiber laser mode locked with a carbon nanotube absorber. (**b**) Optical spectrum. (**c**) Autocorrelation trace [178]. (**d**) Experimental setup of the 3.5 μm mode–locked fiber laser based on SWCNT. (**e**) Optical spectrum. (**f**) Pulse train [181]. (**g**) Experimental setup of the passively mode–locked THDFL based on Bi_2_Te_3_. (**h**) Optical spectrum. (**i**) Autocorrelation trace [185]. (**j**) Configuration of passively mode–locked TDFL incorporating MoO_3_–SA in the ring cavity. (**k**) Optical spectrum. (**l**) Autocorrelation trace [192]. (**m**) The schematic of the ring–cavity Tm^3+^–Ho^3+^–codoped fiber laser system based on Ti_3_C_2_T_x_. (**n**) Optical spectrum. (**o**) Autocorrelation trace [197].

CNTs are 1D nanocrystalline graphite materials with a uniform cylindrical structure and a high aspect ratio. They can be categorized into multiple types according to the quantity of tube walls, specifically single-walled carbon nanotubes (SWCNTs), double-walled carbon nanotubes, and multi-walled carbon nanotubes (MWCNTs). As one of the earliest nanomaterials employed for passive ML, SWCNTs exhibit high third-order nonlinear polarizability and an ultrafast recovery time, which consists of a rapid intraband carrier relaxation time ranging from 0.3 to 1.2 ps and a slow recombination process lasting from 5 to 20 ps [176]. In 2004, CNT SA was first proposed by Set et al. for passively mode-locked laser [39]. In 2006, a SWCNTs–polyvinylalcohol nanocomposite was set in a micro gap between a pair of fiber end facets in EDF laser to achieve 178 fs ML pulses with a repetition rate of 22.8 MHz at 1.56 μm [177]. In 2008, a SWCNT polymer film as a SA was first employed in a 1.93 μm TDF laser to achieve ultrashort pulse of approximately 1.32 ps [178]. The cavity schematic, output spectrum and autocorrelation trace are shown in Figure 8a–c. In 2014, the shortest pulses of 90 fs, to our knowledge, were achieved in a diode-pumped Yb:CLNGG laser by applying SWCNTs SAs for the first time [179]. Moreover, the superior thermal conductivities of CNTs guarantee intrinsic high-power handling. In 2015, a high power Nd:YVO_4_ ML laser operating at 1064 nm was demonstrated by using SWCNT SAs [180]. The maximum output power up to 2.7 W was obtained with a 167 MHz repetition rate and 3.1 ps pulse duration. The calculated pulse energy and peak power are 16.1 nJ and 5.2 kW. As of 2022, Wei et al. realized ML pulse output in a 3.5 μm Er-doped fluoride fiber laser, compressing the output pulse width to 1.66 ps at a repetition frequency of 25.2 MHz [181]. It conducted a comprehensive verification of the nonlinear optical modulation capability of CNTs in the mid-infrared band. The cavity schematic, output spectrum and autocorrelation trace are shown in Figure 8d–f. However, the difficulties in chirality and tube-diameter control of CNT restricts its application as SA at longer working wavelength.

TIs have emerged as another significant LDM subsequent to graphene, attributed to their distinctive Dirac surface states [182]. They act as insulators in their inner portion, but gapless conducting states appear on their surfaces. The combination of the small bandgap bulk (0.2~0.3 eV) and the gapless surface enable TIs to possess an ultra-broad bandwidth of saturable absorption operation. In addition, TIs demonstrate an ultrashort phonon-induced carrier lifetime on the order of several picoseconds, indicating their potential application in ultrafast ML lasers. TIs were used as SAs for demonstrating an ultrafast fiber laser in 2012. Zhao et al. reported a Bi_2_Te_3_ SA exhibiting very-high-modulation-depth (up to 95%) and used as a passive mode locker for ultrafast pulse formation at the telecommunication band [34]. In 2013, a Bi_2_Se_3_ SA was employed for the operation of a bulk solid-state laser [183]. In 2014, the operating wavelength of TIs was successfully extended to 2 μm. A Bi_2_Te_3_ layer with a thickness of approximately 30 μm on a side-polished optical fiber platform (with a modulation depth of approximately 20.6%) was employed to generate stable, ultrafast pulses with a temporal width of approximately 795 fs at a wavelength of 1935 nm from a thulium/holmium co-doped fiber ring cavity. This experimental demonstration confirms that TI SAs can readily be used as an ultrafast mode-locker for 2-μm lasers [184]. Yin et al. integrated Bi_2_Te_3_ nanosheets onto a micro-nanofiber through optical deposition and achieved soliton ML with a 1.26 ps ultrashort pulse in 2 μm thulium/holmium co-doped fiber laser [185]. The cavity schematic, output spectrum and autocorrelation trace are shown in Figure 8g–i. In 2016, a fiber-taper TI SA was fabricated using the PLD method for the first time, it modulated 70 fs pulses at 1542 nm by hybridizing nonlinear polarization evolution (NPE) [186]. In 2017, a Cd_3_As_2_ SA with a relaxation time of 0.5 ps (2% Cr doping concentration) was used for the generation of a stable ML operation at a center wavelength of 2860 nm. The study shows robust and effective tuning of the scattering channels of Dirac fermions, opening up the long-sought parameter space crucial for the development of compact and high-performance mid-infrared ultrafast sources [187]. In 2022, magnetron-sputtered GaSb thin film was used for pulse generation at 1559 nm, 1902 nm and 2783 nm [188]. These results indicate that TIs have great potential for nonlinear optical applications with a wide operating wavelength range.

In view of their substantial third-order optical nonlinearities, rapid recovery time on the picosecond scale, and adjustable band-gap energy, TMOs have emerged as a novel research avenue in the SA domain in recent years. In 2016, Fe_3_O_4_ nanoparticles (FONPs) exhibited a modulation depth of 8.2% and were employed in a stable passive QS EDF laser. An output pulse energy of 23.76 nJ, a repetition rate of 33.3 kHz, and a pulse width of 3.2 µs were achieved [189]. In 2017, Mohd Rusdi et al. initially utilized a titanium dioxide (TiO_2_) film as a SA in a 1.97 μm Tm^3+^Ho^3+^co-doped fiber laser to achieve passive ML, and commenced research on TMOs for 2 μm ultrafast lasers [190]. In 2019, FONPs were deposited on an Au mirror and utilized for QS in a tunable mid-infrared fiber laser with a tunable range of 2812.4~3031.6 nm. This demonstration suggests that TMOs are a promising broadband saturable absorption material for mid-infrared operation [191]. In 2022, Ahmed et al. coated micrometer-scale molybdenum trioxide (MoO_3_) particles compounded with a polymer onto a micro-nanofiber and utilized the evanescent field effect to augment the light–matter interaction [192]. They successfully achieved stable ML in a 1.94 μm TDF laser, resulting in an ultrashort pulse output of 1.22 ps, as shown in Figure 8j–l. The device also demonstrated a high optical damage threshold surpassing 19.34 GW/cm^2^, offering a novel material alternative for the practical realization of high-power ultrafast lasers.

MXenes, as a kind of newly emerging category of 2D transition metal carbides/nitrides, have exhibited significant potential in ultrafast photonics owing to their metallic conductivity, adjustable surface functional groups, and broadband nonlinear optical response. The general formula for MXene is M_n+1_X_n_T_x_, where M refers to transition metals (Ti, Sc, Hf, Zr, Nb, V, Cr, Mo, Ta, etc.), X stands for C, N, or B, and T_x_ is an element from the 3rd or 4th main group (O, OH, F, etc.), where x denotes the number of terminal groups. MXenes exhibit strong saturable absorption behaviors, high carrier mobility and great bandgap tunability. Furthermore, the outstanding mechanical performance, thermoelectric properties, and environmental stability under humid conditions of MXenes materials render them particularly suitable as SAs for pulse modulation applications. The first study of the mode-locked fiber laser using MXenes as the SA was reported by Jhon et al. in 2017 [193]. The uniqueness of Ti_3_C_2_T_x_ lies in its notably small bandgap of less than 0.2 eV, which can be utilized for broadband pulsed laser generation across the visible to mid-infrared wavelengths (e.g., 607, 639, 721, 1066, and 1555 nm) [194,195]. In 2020, Wang et al. conducted a systematic investigation into the broadband nonlinear optical properties of Ti_3_C_2_T_x_ and applied it to ML and QS fiber lasers at 1.55 μm and 2.8 μm [196]. In 2021, Jhon et al. successfully generated a ML pulse with a pulse width of 897 fs in a 1.9 µm infrared fiber laser using a multilayer Ti_3_C_2_T_x_ SA [197]. The cavity schematic, output spectrum and autocorrelation trace are shown in Figure 8m–o. These studies comprehensively illustrate the unique advantages of MXenes as broadband SAs. Subsequently, a Nb_2_C material, a member of the MXenes family, was measured to have an ultrafast relaxation time of 37.43 fs and a slow relaxation time of 0.57 ps [198]. The Nb_2_C SA was coated onto a tapered fiber to facilitate the interaction of the evanescent field, and it generated a soliton pulse with a pulse duration of 0.77 ps at the central wavelength of 1559 nm [199]. The research findings offer a potential avenue for the development of a novel device using Nb_2_C or other types of MXenes for high-performance ML lasers. As shown in Table 4, ultrafast lasers incorporating SAs based on various MXenes materials have been reported, and they demonstrate remarkable laser performance.

**Table 4:** Performance summary of passively modulated lasers based on other absorbers. λ_0_ is the center wavelength, τ_p_ is the pulse width, f_rep_ is the repetition frequency, E_p_ is the pulse energy, P_ave_ is the average output power.

SA	Gain Medium	λ_0_ (nm)	τ_p_ (ps)	f_rep_ (KHz)	E_p_ (nJ)	P_ave_ (mW)	Thickness	References
CNT	Yb^3+^: CLNGG	1049	0.09	83,000	-	90	single-wall	[179]
CNT	Nd^3+^: YVO_4_	1064	3.1	167,000	16.1	2700	single-wall	[180]
CNT	Er^3+^ silica fiber	1560	0.178	22,800	0.068	1.55	35 μm	[177]
CNT	Tm^3+^ silica fiber	1870	0.45	45,000	0.4	18	10 μm	[200]
CNT	Tm^3+^ silica fiber	1895	0.56	-	1.66	99.4	50 μm	[201]
CNT	Tm^3+^ silica fiber	1928	0.501	-	0.5	-	100 nm	[202]
CNT	Tm^3+^ silica fiber	1927	0.152	25,760	0.19	-	30 μm	[203]
CNT	Tm^3+^ silica fiber	1932	1.32	37,000	-	3.4	single-wall	[178]
CNT	Tm^3+^ silica fiber	1947	2.3	6710	0.45	3	single-wall	[204]
CNT	Tm^3+^ silica fiber	1941.6	3.7	17,640	-	-	single-wall	[205]
CNT	Tm^3+^ silica fiber	1950	0.972	21,050	0.109	2.3	single-wall	[206]
CNT	Tm^3+^/Ho^3+^ silica fiber	1860~2010	2.38~2.83	18,400	-	-	20 μm	[207]
CNT	Ho^3+^ silica fiber	2079	0.683	54,520	0.376	20.5	400 nm	[208]
CNT	Er^3+^: ZBLAN	3470	1.66	25,200	0.98	25	1 μm	[181]
Bi_2_Te_3_	Tm^3+^/Ho^3+^ silica fiber	1909.5	1.26	21,500	-	2.3	20 nm	[185]
Bi_2_Te_3_	Tm^3+^/Ho^3+^ silica fiber	1935	0.795	27,900	-	20	30 μm	[184]
Bi_2_Se_3_	Tm^3+^/Ho^3+^ silica fiber	1912	0.853	18,370	-	-	15 μm	[209]
Cd_3_As_2_	Ho^3+^/Pr^3+^: ZBLAN	2864.3	6.3	14,280	-	-	400 nm	[187]
CoSb_3_	Tm^3+^/Ho^3+^ silica fiber	1913	0.838	16,930	-	-	6 μm	[210]
Sb_2_Se_3_	Er^3+^ silica fiber	1560	2.1 × 10^6^	62.5	2.104	1.15	3~4 nm	[211]
Sb_2_Se_3_	Er^3+^ silica fiber	1562	16,500	3540	0.9	3.1	25 μm	[212]
Sb_2_Se_3_	Er^3+^ silica fiber	1562.4	0.63	22,600	0.0156	-	4 nm	[213]
Sb_2_Se_3_	Tm^3+^ silica fiber	1961	0.89	22,360	-	-	125 nm	[214]
Sb_2_Te_3_	Er^3+^ silica fiber	1542	0.07	95,400	-	63	-	[186]
Sb_2_Te_3_	Er^3+^ silica fiber	1558	1.9	3750	-	-	80 nm	[215]
Sb_2_Te_3_	Er^3+^ silica fiber	1565.5	0.452	20,100	0.091	1.82	18 nm	[216]
Sb_2_Te_3_	Tm^3+^: YAG	2012.6	3.82 × 10^5^	56.67	4800	272	70 nm	[217]
Sb_2_Te_3_	Ho^3+^ silica fiber	2081.9	1.85	13,410	-	0.77	-	[218]
Bi_2_Te_3_	Er^3+^ silica fiber	1560.8	0.286	18,550	0.027	0.5	670 nm	[219]
Bi_2_Se_3_	Er^3+^ silica fiber	1564.6	1.57	1210	-	-	-	[220]
GaSb	Er^3+^ silica fiber	1559	0.308	15,280	0.98	17.82	103 nm	[187]
	Tm^3+^ silica fiber	1902	0.585	19,720	1.37	64.69		
	Er^3+^: ZBLAN	2783	4.99 × 10^5^	137	3070	420		
Te	Er^3+^: ZBLAN	2782.3	4.57 × 10^5^	116.98	3050	357	40.7 nm	[221]
TiO_2_	Tm^3+^/Ho^3+^ silica fiber	1979	10.29	9000	1.66	-	30 μm	[189]
TiO_2_	Tm^3+^ silica fiber	1925.6	1.5	11,530	11	126.8	-	[222]
Fe_3_O_4_	Er^3+^ silica fiber	1560	3.2 × 10^6^	33.3	24	0.8	20 μm	[188]
Fe_3_O_4_	Dy^3+^: ZBLAN	2931.2	1.25 × 10^6^	123	900	111	-	[190]
Co_3_O_4_	Tm^3+^ silica fiber	1958	1.39	11,360	0.023	0.26	-	[223]
ZnO	Tm^3+^ silica fiber	1945	1.395	11,360	0.056	0.64	45 μm	[224]
MoO_x_	Tm^3+^/Ho^3+^ silica fiber	1937.1	1.64	11,500	-	1.3	-	[225]
WO_3_	Tm^3+^ silica fiber	1941.4	1.22	10,840	0.68	-	-	[191]
BiVO_4_	Er^3+^ silica fiber	1563.3	2.67 × 10^6^	76.28	330.76	25.23	-	[226]
Ti_3_C_2_T_x_	Yb^3+^ silica fiber	1065.9	480	18,960	-	-	-	[193]
	Er^3+^ silica fiber	1555	0.159	7280	-	-		
Ti_3_C_2_T_x_	Tm^3+^/Ho^3+^ silica fiber	1914	0.897	16,770	-	-	80~800 nm	[196]
Ti_3_C_2_T_x_	Tm^3+^ silica fiber	1891.8	2.18	5970	-	57.6	3~12 nm	[195]
	Er^3+^: ZBLAN	2788	6.4 × 10^5^	122.9	1850	227		
Ti_2_C	Tm^3+^/Ho^3+^ silica fiber	1933.8	1.655	11,560	-	50.9	-	[227]
Ti_2_AlC		1894.5	1.382	12,400	-	20.4	5 μm	
Ti_3_CN	Er^3+^ silica fiber	1557	0.66	15,400	-	0.05	4~6 μm	[192]
Nb_2_C	Er^3+^ silica fiber	1559	0.77	14,120	0.213	3.04	-	[198]
V_2_C	Tm^3+^/Ho^3+^ silica fiber	1937	1.68	11,520	0.235	-	-	[228]
V_2_C	Tm^3+^/Ho^3+^ silica fiber	1900	0.843	18,290	-	-	11 nm	[31]
Ni-MOF	Er^3+^ silica fiber	1563	0.384	17,000	-	-	4.2 nm	[35]
	Tm^3+^ silica fiber	1882	1.3	13,900	-	-		

### 3.5. Heterostructures

As the application of individual LDMs deepens, their respective limitations gradually become apparent. For instance, graphene exhibits an inadequate modulation depth, BP demonstrates poor stability, and TMDs have a relatively long recovery time. To overcome these limitations, researchers have proposed the construction of heterostructures through the vertical stacking of various 2D materials via van der Waals forces. They utilize the interlayer coupling effects of heterostructures to facilitate electron transfer and interband transitions, thereby achieving optical synergistic effects [229]. This approach offers a novel concept for material design in the development of high-performance ultrafast lasers.

**Figure 9:**
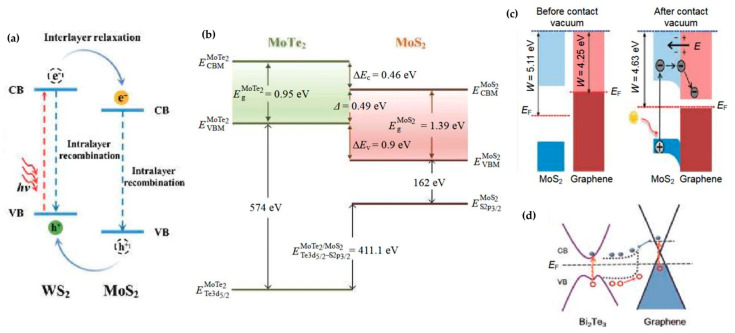
Illustration of the energy band and carrier mobility diagrams of several heterostructures [234]. (**a**) MoS_2_/WS_2_ [230]. (**b**) MoTe_2_/MoS_2_ [231]. (**c**) MoS_2_/graphene [232]. (**d**) Bi_2_Te_3_/graphene [233].

Figure 9 illustrates the energy band diagrams and carrier transport schemes of several heterostructures. Figure 9a depicts the interlayer relaxation process of photogenerated carriers within a WS_2_/MoS_2_ Type-II heterostructure [230], suggesting that the Type-II band alignment facilitates the spatial segregation of photogenerated electrons and holes. This enables sub-picosecond ultrafast responses by modulating carrier relaxation dynamics to support femtosecond ultrashort pulse output. Meanwhile, it resolves the self-starting issue of passive ML by optimizing the modulation depth. Additionally, it suppresses non-saturable losses resulting from intralayer recombination to enhance optical conversion efficiency. Figure 9b presents the quantitatively calibrated band offset and bandgap parameters of the MoTe_2_/MoS_2_ heterostructure [231]. The absorption edge and nonlinear optical properties can be customized and controlled by the regulate of heterojunction. This facilitates the expansion of the working bandwidth of saturable absorption through the combination of materials with different bandgaps, which is appropriate for accommodating lasers of diverse wavelengths. Figure 9c depicts the interfacial band evolution and charge transfer process in the MoS_2_/graphene contact prior to and subsequent to contact formation [232]. The semiconductor–semimetal heterostructures can address the intrinsic conflict between the “ultrafast response speed” and the “high damage threshold” in conventional SAs materials. Photogenerated carriers are capable of undergoing ultrafast transfer at the interface within a sub-hundred-femtosecond timescale. By integrating the ultrafast relaxation characteristic of graphene with the high optical damage threshold of semiconductor materials, it is possible to mitigate intracavity thermal effects, thereby significantly enhancing the output power ceiling and long-term operational stability of the laser. Figure 9d depicts the band structure and carrier transport behavior of a Bi_2_Te_3_/graphene heterostructure [233], indicating that the working wavelength of SAs can be further expanded to special bands. An ultra-broadband, low-loss saturable absorption SA was realized by utilizing the topological surface states of topological insulators and the Dirac-cone band matching with graphene. Consequently, it significantly enhances the wavelength tuning capability and broadens the application scenarios of passively modulated lasers.

**Figure 10:**
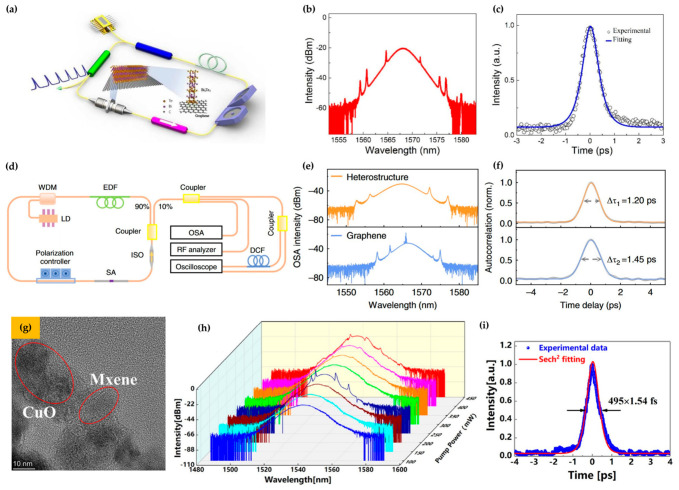
The performance of passively modulated lasers based on heterostructure. (**a**) The configuration of the laser cavity based on graphene/Bi_2_Te_3_. (**b**) Optical spectrum. (**c**) Autocorrelation trace [235]. (**d**) Schematic of the mode–locked all–fiber laser and measurement system. (**e**) Spectra of the output lasers with the bare graphene–SA and heterostructure–SA. (**f**) Autocorrelation traces [237]. (**g**) SEM image of Ti_3_C_2_T_x_/CuO composite. (**h**) Optical spectrum. (**i**) Autocorrelation trace [241].

During the initial exploration phase, research on heterostructure SAs primarily focused on graphene-TIs heterostructures. In 2015, Mu et al. first fabricated a graphene/Bi_2_Te_3_ heterostructure SA with controllable optical properties by two-step CVD method [235]. By regulating the coverage of Bi_2_Te_3_ nanoplatelets, it demonstrates a larger modulation depth (ranging from 14.7% to 50.1%) in comparison to pure graphene and faster carrier dynamics (spanning from 191 fs to 296 fs) in comparison to pure Bi_2_Te_3_. The laser cavity, output spectrum and pulse trace are shown in Figure 10a–c. The research delivers a new tunable photonics material which may find wide applications for pulse laser generation or signal processing.

Subsequently, graphene-based heterostructure SAs achieved comprehensive expansion in terms of material systems, modulation depth, operating wavelength, and stability. Gao et al. utilized a graphene/BN heterostructure SA in a 2 μm Tm^3+^:YAP passive QS laser. A minimum pulse width of 607 ns with an average output power of 3.67 W was obtained [236]. In comparison with single-layer graphene SA, the pulse width was narrower, the output power was higher, and the thermal stability was notably enhanced. This approach addressed the issues of inadequate modulation depth and severe heat accumulation intrinsic to single-layer graphene SAs. Shao et al. devised a MoS_2_/BN/graphene/BN/MoS_2_ SA and attained an ultrashort pulse output of 1.2 ps in a 1.5 μm all-fiber mode-locked laser [237]. The polarization tolerance of single-pulse ML was elevated from 20% (pure graphene) to 85%, significantly augmenting the operational stability of ML lasers. The schematic diagram of the cavity, the output spectrum, and the autocorrelation trace are presented in Figure 10d–f. Wang et al. fabricated a tellurium (Te)/graphene heterostructure SA and achieved stable ML (with a pulse width of 12.18 ps) in a 3 μm fiber laser [238]. This study demonstrated that heterojunctions are an effective method to improve nonlinearity, which is a promising approach to achieve high-efficiency pulsed laser.

Within non-graphene-based heterostructure systems, combinatorial innovations among TMDs, TMOs, MXenes, and ITs have emerged as research focal points. In 2018, it was confirmed that MoS_2_-Sb_2_Te_2_-MoS_2_ heterostructure materials exhibit a large modulation depth of 64.17% and can withstand high power during the generation of ultrashort pulses [239]. The new type of TMDs/TIs provides a promising solution for the generation of stable high-energy ultrashort pulses. In 2019, Liu et al. employed the magnetron sputtering deposition technique to fabricate a MoS_2_/WS_2_ heterostructure coated on the tapered fiber [240]. In comparison with monolayer MoS_2_, this heterostructure demonstrates a smaller band gap and higher carrier mobility, which may lead to a greater modulation depth. Consequently, a generated pulse duration of 154 fs was achieved by utilizing the prepared SA in a EDF laser. Pang et al. in 2025 further developed a Ti_3_C_2_T_x_/CuO heterostructure SA and demonstrated both conventional soliton output of 495 fs and dissipative soliton output of 22 ps in a 1.5 μm fiber laser [241], enriching the operational modes of MXene-based heterostructures. The SEM image of Ti_3_C_2_T_x_/CuO composite, output spectrum and autocorrelation trace are shown in Figure 10g–i. Compared to single materials, heterojunctions based on TMOs exhibit significantly improved stability, further enhancing the pulse energy and peak power of lasers [242]. Later, the unique nonlinear optical properties of multifarious heterojunctions were verified, providing an optimized method for the saturable absorption properties of materials [241,242,243,244,245,246,247,248,249,250].

In general, heterostructure SAs have fundamentally overcome the performance bottlenecks of single 2D material SAs through the performance synergy of multiple materials and have emerged as the core development direction in the field of passively modulated ultrafast lasers. Table 5 summarizes the achievements of passively modulated lasers based on heterostructure SAs. The subsequent research focuses on the standardization and upscaling of heterostructure fabrication processes, the atomic-level precise regulation of interfacial properties, the reduction of non-saturable losses, and the improvement of optical damage thresholds. It will propel heterostructure SAs from basic laboratory research towards industrialized and commercial practical applications [237].

### 3.6. Summary

**Figure 11:**
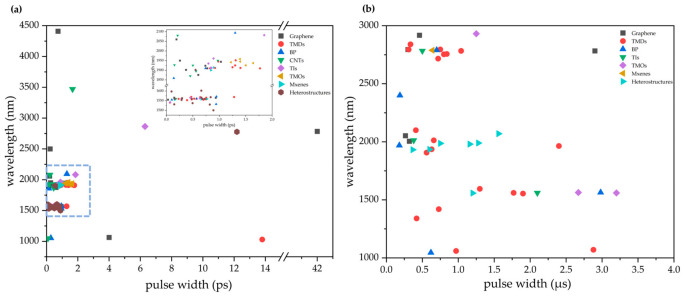
(**a**) Passive ML lasers based on LDMs, the illustration in the picture is a magnification of the part with the blue dashed box. (**b**) Passive QS lasers based on LDMs.

Figure 11a compiles the center wavelength and pulse duration of passive ML lasers employing different LDM SAs, including graphene, TMDs, BP, CNTs, TIs, TMOs, MXenes, and heterostructures. In accordance with the figure, graphene-based ML lasers exhibit an ultra-broadband operation from ~1 μm to 4.4 μm, with pulse widths down to tens of femtoseconds (e.g., 29 fs), benefiting from its zero-bandgap structure and ultrafast carrier dynamics (initial relaxation time ~70 fs). TMDs (e.g., MoS_2_, WS_2_, MoSe_2_, WSe_2_) cover a wide near- to mid-infrared range (~1 μm to ~2.8 μm) and achieve pulse durations as short as ~67 fs (WS_2_) or ~154 fs (MoS_2_/WS_2_ heterostructure), owing to their layer-dependent direct bandgap and strong nonlinear absorption. Black phosphorus (BP)-based lasers demonstrate broad tunability from ~1 μm to ~3.5 μm with ultrafast pulse widths reaching ~102 fs, attributed to its direct bandgap (0.3–2.0 eV) and large modulation depth (~15–27%). CNTs, TIs (e.g., Bi_2_Te_3_, Bi_2_Se_3_, Sb_2_Te_3_), TMOs (e.g., MoO_3_, TiO_2_), and MXenes (e.g., Ti_3_C_2_Tₓ) also contribute to sub-picosecond and few-picosecond pulse generation across the 1.5–3.5 μm region, with CNTs showing excellent thermal stability and TIs offering ultrahigh modulation depth (up to 95%). Heterostructures (e.g., graphene/Bi_2_Te_3_, WS_2_/MoS_2_, Ti_3_C_2_T_x_/CuO) further extend the wavelength coverage and improve pulse performance through synergistic band alignment and interfacial charge transfer, achieving pulse widths as short as ~154 fs. Overall, the data confirm that LDM SAs enable compact, broadband, and ultrafast mode-locked lasers spanning from the near-infrared to mid-infrared.

Figure 11b presents the center wavelength and pulse duration of passive QS lasers utilizing different LDM SAs. In accordance with the figure, graphene-based QS lasers operate over a broad spectral range from ~1 μm to 2.8 μm, with pulse durations typically in the sub-microsecond to microsecond regime (e.g., 300 ns at 2.8 μm), benefiting from its high damage threshold and broadband modulation capability. TMDs (e.g., MoS_2_, WS_2_, MoSe_2_, WSe_2_) and BP also enable stable QS operation in the near- to mid-infrared (e.g., 2.8 μm for MoS_2_, 3.5 μm for BP), with pulse widths ranging from hundreds of nanoseconds to a few microseconds. CNTs, TIs, TMOs (e.g., Fe_3_O_4_, TiO_2_), and MXenes (e.g., Ti_3_C_2_T_x_) further demonstrate QS operation at wavelengths extending to ~3 μm, with pulse energies reaching tens of microjoules and average powers at the watt level. Heterostructures (e.g., Te/graphene, WSe_2_/CuO) show enhanced stability and modulation depth, enabling high-energy QS pulses in the mid-infrared. The compiled results underline that LDM SAs are highly effective for passive QS, offering flexible wavelength tunability, simple cavity designs, and robust pulse generation across the 1–4 μm spectral region, which is critical for applications requiring high pulse energy and moderate pulse width.

## 4. Synthesis Methods for LDM SAs

**Figure 12:**
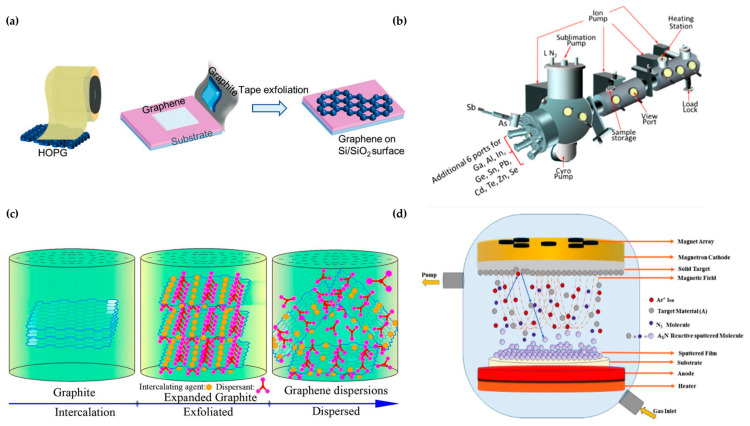
(**a**) Schematic diagram of the process of mechanical exfoliation of graphene [258]. (**b**) Schematic showing the single-chamber MBE system and its flexible arrangement of effusion cells [259]. (**c**) Microstructure diagram of graphene exfoliation and dispersion by stripping graphite in liquid phase [260]. (**d**) Schematic diagram of magnetron sputtering [261].

The synthetic techniques for the LDM SAs are classified into top-down and bottom-up approaches. The top-down exfoliation methods comprise liquid-phase exfoliation (LPE), mechanical exfoliation (ME), laser etching, aqueous acid etching, and electrochemical exfoliation, where single-layer or new-layer nanosheets are separated from bulk materials by violating the van der Waals force between layers. Bottom-up methods include molecular beam epitaxy (MBE), magnetron sputtering, PLD and CVD, where high-quality 2D materials in atomic layer scale are effectively synthesized by explicitly adjusting the chemical reactions among solid precursors. Figure 12 shown the schematic diagram of most common synthesis methods.

The ME technique is commonly used in the manufacturing of 2D layered nanomaterials (e.g., graphene, MoS_2_, WS_2_, SnS_2_, BP). High-quality mono- and few-layer materials can be obtained by using adhesive tape to overcome interlayer van der Waals forces. Since its first application in graphene in 2014, this method has been extensively employed for the preparation of 2D SAs materials owing to its simplicity, flexibility, and cost-effectiveness. Nevertheless, it does possess certain drawbacks. For instance, it is unable to synthesize large-area single-layer materials, and the thickness is non-uniform and uncontrollable. LPE is capable of eliminating interlayer forces through ultrasonication and exfoliating LDMs from their bulk (or aggregation) state in liquid media. This methodology can also be integrated with other approaches, such as ion exchange and ion intercalation. Consequently, this synthesis method has been extensively applied throughout the entire LDMs system (e.g., graphene, BP, TMDs, MXenes, CNTs, TMOs, TIs, MOFs). Although this approach facilitates the regulation of the size of nanomaterials and produces highly concentrated dispersions by adjusting the reducing agent, pH, or surfactant, it yields nanosheets with wide thickness distributions and may introduce solvent residues or structural defects.

In general, the introduction of impurities and defects also constitutes the common drawback of all top-down preparation methods. In addition, the uncontrollable scale and random thickness of few-layered materials acquired using the top-down techniques are counterproductive to the efficiency of an SA. By contrast, the bottom-up approach uses physical or chemical methods to deposit or build up LDMs. During this growth process, precise control over their size and morphology can be achieved by regulating the precursor and growth conditions.

Magnetron sputtering, as a physical vapor deposition (PVD) technique, achieves the deposition of materials into thin films by bombarding a target with ions produced by glow discharge. This method has currently been used to prepare 1D and 2D thin films (e.g., carbon-based, sulfide-based, nitride-based, and heterostructures) with precisely controlled thickness. However, the equipment cost associated with this technique is comparatively high, and the sputtering process may lead to stoichiometric deviations. The other two PVD growth techniques are PLD and MED. PLD is a methodology that employs high-energy-density lasers to irradiate target materials, enabling the deposition of the ejected material onto a substrate for the fabrication of thin films. MBE enables layer-by-layer growth with atomic-level precision under ultrahigh vacuum by precisely controlling the beam current and energy of the source molecules. Both preparation methods produce nano-films of extremely high purity and perfect lattice quality, which are well-suited for growing high-quality low-dimensional quantum dots, multicomponent thin films, heterojunctions, and other nanostructures. Nevertheless, their applications in the field of nanomaterial preparation are still limited due to the high costs of equipment and the difficulties in technical control.

**Figure 13:**
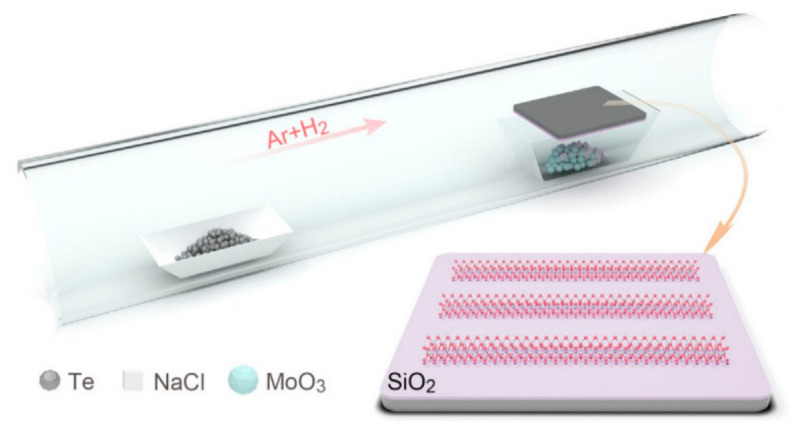
Schematic diagram of the CVD setup for synthesis of aligned MoTe_2_ nanoribbons [262].

CVD is based on the thermodynamics of surface chemical reactions. Gaseous precursors undergo decomposition, adsorption, nucleation and growth on the surface of a high-temperature substrate. In the case of 2D materials, the growth process adheres to a self-limiting mechanism. Once a single layer is formed, the active sites on the surface become passivated. Subsequently, the growth of additional layers necessitates surmounting a higher energy barrier, which enables precise regulation of the layer number. In comparison to top-down exfoliation techniques, CVD facilitates the controllable growth of large-area, high-quality thin films with accurate layer-number regulation, and has emerged as the predominant growth approach for LDMs and heterojunctions. Figure 13 shows the schematic diagram of the CVD setup for synthesis of aligned MoTe_2_ nanoribbons [262].

However, the current routes (containing two steps: chemical vapor deposition and spin coating) for constructing LDM SAs are suffering from limited flexibility in substrate choice and the introduction of impurities during the transfer process or due to molecular functional groups. This affects the broadband absorption parameters of LDM SAs, such as non-saturated loss, ultrafast optical response, modulation depth, stability, and thermal tolerance.

**Figure 14:**
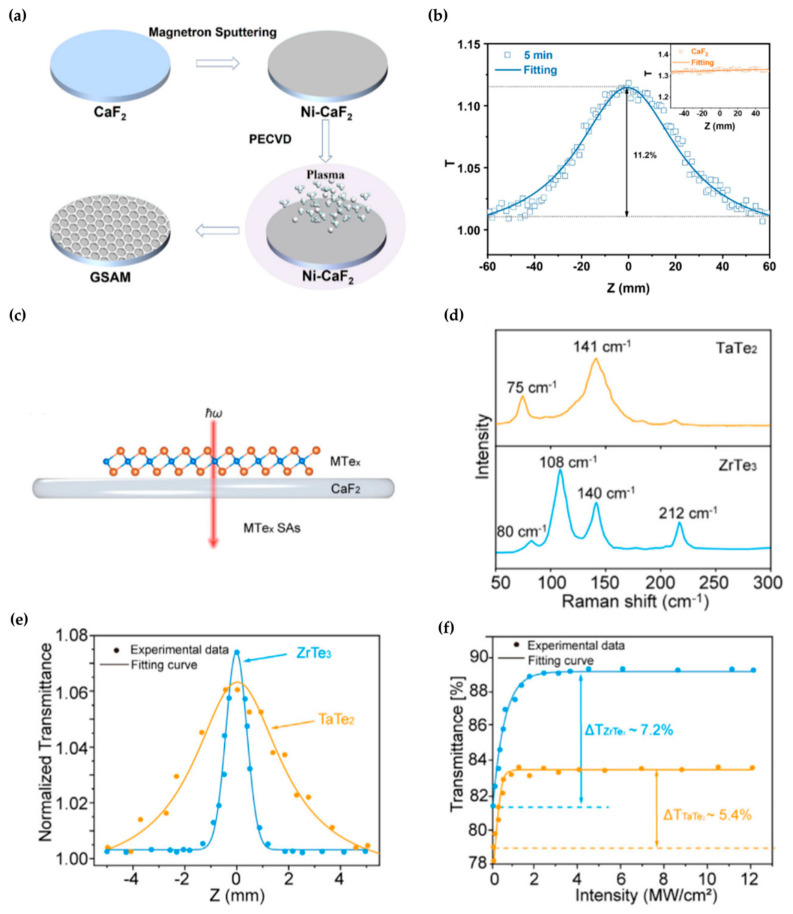
(**a**) Schematic illustration of fabricating graphene saturable absorber mirror. (**b**) Open aperture Z–scan curve of the GSAM [63]. (**c**) Schematic of TMT–SAs. (**d**) Raman spectra of TaTe_2_– and ZrTe_3_–SAs. (**e**) Open–aperture (OA) Z–scan curves of TaTe_2_– and ZrTe_3_–SAs. (**f**) Nonlinear absorption curves of TaTe_2_– and ZrTe_3_–SAs [112].

In recent years, our research group has been investigating methods for direct CVD growth on substrates. As of 2022, we have demonstrated a high-quality graphene saturable absorber mirror (GSAM) grown directly on a calcium fluoride (CaF_2_) substrate by a low-temperature plasma enhanced chemical vapor deposition (PECVD) method for mid-infrared pulse modulation [63]. Figure 14a shows the process of PECVD synthesis and material characterization. The controllable growth of a high-quality graphene film on the nickel-modified CaF_2_ substrate was realized by adjusting the growth time and hydrocarbon ratio. This method circumvents wet-coating procedures in various previously reported SAs, thereby mitigating the impurity-induced performance degradation. Consequently, the GSAM exhibits excellent nonlinear optical absorption and operates a high-efficiency and high-peak power QS laser at 2.8 μm.

In 2025, our group fabricated 2D transition metal telluride (TMT) SAs using the PECVD method [112]. This approach involves the direct in situ growth of large-area, high-quality 2D ZrTe_3_ and TaTe_2_ films on CaF_2_ substrates as SA mirrors, followed by oxygen plasma passivation. Passivation serves to effectively protect the SA material from widespread atmospheric corrosion and laser-induced damage. The images of TMT-SAs are shown in Figure 14c–f. TaTe_2_ and ZrTe_3_ SAs exhibit strong nonlinear optical absorption properties, with modulation depths of 5.4% and 7.2%, respectively. Remarkably, the as-synthesized 2D TMT-SAs retain excellent long-term stability, which are capable of generating stable Q-switched MIR pulses. Our strategy paves a way for developing high-quality and stable GSAMs for industrial applications of pulsed mid-infrared lasers.

## 5. Conclusions and Outlooks

This paper has systematically reviewed the research progress of near/mid-infrared pulsed lasers based on LDM SAs, encompassing the fundamental principles of saturable absorption and passive modulation, the nonlinear optical properties of various LDMs (graphene, TMDs, BP, CNTs, TIs, TMOs, MXenes), device fabrication methods, and their application results in Q-switched and mode-locked lasers. Overall, significant advances in pulsed lasers based on LMDs SAs have been achieved, which focus on key performance metrics, including center wavelength (from the 400 nm range to 4.4 μm), pulse width (from sub-microsecond to tens of femtoseconds), repetition rate (from kHz to hundreds of MHz), pulsed energy (from sub-nanojoule to dozens of microjoules), and output peak power (from watt to kilowatt level). They provide compact, efficient, and ultrafast laser source solutions for applications in biomedicine, atmospheric remote sensing, materials processing, and nonlinear optics. Although LDM SAs have achieved notable advancements in the domain of ultrafast laser pulse modulation, a number of core challenges persist before the complete realization of the transition from laboratory research to industrial and practical applications. In view of the current development trends within the field, future research is anticipated to attain breakthroughs in the subsequent directions.

The subsequent crucial step to be addressed is the exploration of a highly stable and batch fabricable preparation technology for LDM SAs. Environmental stability (especially for sensitive materials like BP and MXenes) and device consistency are major obstacles to practical application. Standardizing scalable fabrication processes, such as CVD and PECVD, is crucial to achieving a low-cost and reproducible manufacturing of high-performance SAs. Simultaneously, future strategies ought to encompass the in situ passivation, atomic layer deposition encapsulation, polymer composites, and inorganic/organic multilayer coating approaches. These approaches are anticipated to significantly enhance the material’s resistance to moisture and oxidation, all the while preserving its nonlinear performance.

At the laser system level, it is of great significance to promote the integration of all-fiber, all-solid-state, and photonic chip technologies. This is conducive to further compressing the pulse width to the sub-hundred femtosecond, increasing the repetition rate, and simultaneously enhancing the single-pulse energy and peak power. At present, the mid-infrared laser still suffers from a shortage of SAs and crucial fiber-optic components (e.g., isolators, couplers, and wavelength division multiplexers). The extensive utilization of free-space SAs exerts a significant influence on the stability and compactness of the system, which constitutes a fundamental device-related bottleneck that requires immediate resolution. Therefore, the mid-infrared LDM SAs need to be monolithically integrated with components such as micro/nano optical fiber, micro/nano optical cavities, waveguides, and fiber Bragg gratings to construct compact, robust, and tunable ultrafast mid-infrared laser sources.

The current maximum operation wavelength demonstrated in LDM SAs is approximately 4.4 μm [67]. Nevertheless, it does not represent the fundamental upper bound, and there is still substantial scope for improvement. The main challenges for extending the wavelength further into the mid-infrared (e.g., 5–10 μm and beyond) include: (i) the intrinsic bandgap limitations of most LDMs, (ii) increased free-carrier absorption and phonon absorption at longer wavelengths, and (iii) the lack of compatible fiber-optic components in this spectral region. The emergence of narrow-bandgap materials (e.g., graphene, TIs, MXenes) and the development of advanced fabrication strategies present promising approaches for the extension of the operating wavelength.

In summary, LDM SAs are undergoing a critical transition from being “material-driven” to “performance-driven” and “application-driven”. Through multi-dimensional advancements of LDM SAs in material innovation, heterostructure engineering, device integration, and advanced fabrication techniques, pulsed lasers are expected to achieve superior performance, enhanced stability, and a higher level of integration in the near future.

## Figures and Tables

**Table 1:** Performance summary of passively modulated lasers based on graphene saturable absorbers. λ_0_ is the center wavelength, τ_p_ is the pulse width, f_rep_ is the repetition frequency, E_p_ is the pulse energy, P_ave_ is the average output power.

SA	Gain Medium	λ_0_ (nm)	τ_p_ (ps)	f_rep_ (KHz)	E_p_ (nJ)	P_ave_ (mW)	Thickness	References
Graphene	Nd^3+^: YAG	1064	4	88,000	-	-	-	[50]
Graphene	Nd^3+^: GdVO_4_	1065	16	43,000	8.4	360	few-layer	[70]
Graphene	Er^3+^ silica fiber	1545	0.088	21,150	0.071	1.5	20.4 nm	[61]
Graphene	Er^3+^ silica fiber	1555	0.029	18,670	2.8	52	10.2~11.9 nm	[62]
Graphene	Er^3+^ silica fiber	1558	0.605	20,800	0.013	0.2	few-layer	[71]
Graphene	Er^3+^ silica fiber	1560	0.98	28,500	0.0275	-	few-layer	[72]
Graphene	Er^3+^ silica fiber	1560	0.174	27,400	0.044	1.2	few-layer	[73]
Graphene	Er^3+^ silica fiber	1563	0.74	119,900	0.1119	-	-	[74]
Graphene	Er^3+^ silica fiber	1564.9	0.758	22,800	0.62	14.1	few-layer	[75]
Graphene	Er^3+^ silica fiber	1565	0.756	1790	-	2	few-layer	[26]
Graphene	Er^3+^ silica fiber	1565	13.8	16,990	10.2	174	few-layer	[59]
Graphene	Er^3+^ silica fiber	1565.3	0.148	101,320	0.025	2.5	12.6 nm	[76]
Graphene	Er^3+^ silica fiber	1566~1570	3.7	3.3~65.9	16.7	34	few-layer	[77]
Graphene	Er^3+^ silica fiber	1576.3	0.415	6840	7.3	-	few-layer	[78]
Graphene	Er^3+^/Tm^3+^ silica fiber	1563	0.7	12,905	24	46.1	0.34 nm	[69]
		1931	1.77	12,905	26	55.9		
Graphene	Tm^3+^ silica fiber	1876	0.603	41,000	-	-	8.2 nm	[79]
Graphene	Tm^3+^/Ho^3+^ silica fiber	1879.4	4.7	7800	-	450	0.34 nm	[80]
Graphene	Tm^3+^ silica fiber	1900	1.9	19,700	-	1.96	0.34 nm	[81]
Graphene	Tm^3+^/Bi^3+^ silica fiber	1902	0.37	16,700	-	1.72	0.34 nm	[53]
Graphene	Tm^3+^ silica fiber	1910	0.773	19,310	6	115	0.34 nm	[82]
Graphene	Tm^3+^ silica fiber	1923.3	0.737	28,250	-	1.21	8.16 nm	[60]
Graphene	Tm^3+^ silica fiber	1940	3.6	6460	0.4	2	-	[52]
Graphene	Er^3+^/Tm^3+^ silica fiber	1565	0.933	20,190	-	0.5	0.68 nm	[55]
		1944	1.03	18,430	-	1.3		
Graphene	Tm^3+^ silica fiber	1945	205	58,870	0.22	13	11.9 nm	[83]
Graphene	Tm^3+^ silica fiber	1950	0.255	23,500	51.5	1210	20.4 nm	[64]
Graphene	Tm^3+^ silica fiber	1953.3	2.1	16,937	0.08	1.41	1.36 nm	[54]
Graphene	Tm^3+^ silica fiber	1983	1.88	11,350	0.3	3.43	few-layer	[84]
Graphene	Tm^3+^ silica fiber	2005	3.2 × 10^5^	280	18,000	5200	0.34 nm	[58]
Graphene	Tm^3+^: CLNGG	2018	0.729	98,700	-	60.2	0.34~0.68 nm	[51]
Graphene	Ho^3+^: YVO_4_	2052.1	2.65 × 10^5^	131.6	16,800	2200	1.02 nm	[85]
Graphene	Ho^3+^ silica fiber	2060	0.19	21,130	2.55	54	-	[67]
Graphene	Cr^3+^: ZnSe	2500	0.226	77,000	-	80	0.34 nm	[56]
Graphene	Er^3+^: ZBLAN	2783	2.9 × 10^6^	37	1670	62	few-layer	[57]
Graphene	Er^3+^: ZBLAN	2784.5	42	25,400	0.7	18	1.36~2.04 nm	[66]
Graphene	Er^3+^: ZBLAN	2795.2	3 × 10^5^	61	-	142	few-layer	[63]
Graphene	Er^3+^: YAP	2918	4.6 × 10^5^	114	5100	1170	0.34 nm	[86]
Graphene	Fe^3+^: ZnSe	4410	0.732	100,000	-	415	0.34 nm	[68]

**Table 5:** Performance summary of passively modulated lasers based on heterostructure absorbers. λ_0_ is the center wavelength, τ_p_ is the pulse width, f_rep_ is the repetition frequency, E_p_ is the pulse energy, P_ave_ is the average output power.

SA	Gain Medium	λ_0_ (nm)	τ_p_ (ps)	f_rep_ (KHz)	E_p_ (nJ)	P_ave_ (mW)	Thickness	References
Te/Se	Er^3+^ silica fiber	1500	0.889	18,500	-	-	25~33 nm	[245]
Graphene/BP	Er^3+^ silica fiber	1529.9	0.82	7430	-	-	4.4~5.4 nm/ 7.9~13.6 nm	[251]
		1531	0.148	7500	-	-		
Ti_3_C_2_T_x_/CuO	Er^3+^ silica fiber	1537	0.495	9210	-	3.87	-	[241]
MoS_2_/Sb_2_Te_3_	Er^3+^ silica fiber	1554	0.286	36,460	-	20	24 nm	[239]
Graphene/WTe_2_	Er^3+^ silica fiber	1558	1.2 × 10^6^	56.8	3.77	3.15	-	[252]
WS_2_/MoS_2_	Yb^3+^ silica fiber	1035.07	0.748	3900	-	6.8	1.69 nm	[243]
	Er^3+^ silica fiber	1558.6	0.682	3900	-	10.32	1.86 nm	
Bi_2_Te_3_/FeTe_2_	Er^3+^ silica fiber	1558.8	0.481	23,000	0.27	6.257	-	[250]
Graphene/Mo_2_C	Er^3+^ silica fiber	1559	0.723	15,320	0.713	10.93	monolayer/ 7.8 nm	[253]
WS_2_/MoS_2_	Er^3+^ silica fiber	1560	0.154	74,672	-	19.8	63 nm	[240]
WS_2_/MoS_2_	Er^3+^ silica fiber	1562.6	0.296	36,460	-	25	132.3 nm	[230]
WSe_2_/MoSe_2_	Er^3+^ silica fiber	1564	1100	4459	0.7	3.1	2.5 nm	[246]
Graphene/ BN/MoS_2_	Er^3+^ silica fiber	1565.8	1.2	13,200	-	-	-	[237]
Ti_3_C_2_T_x_/TiO_2_	Er^3+^ silica fiber	1565.8	0.661	22,750	-	5.964	-	[244]
Graphene/MoS_2_	Er^3+^ silica fiber	1567.2	9.31 × 10^6^	21.9	98.6	2.16	-	[254]
		1571.8	2.2	3470	-	-		
Graphene/Bi_2_Te_3_	Er^3+^ silica fiber	1568	0.837	17,300	0.178	3.07	monolayer/ 8.5 nm	[235]
Graphene/WS_2_	Er^3+^ silica fiber	1593.5	1.41	3630	0.52	1.9	monolayer/ monolayer	[255]
		1601.9	0.66	21,780	-	-		
Nb_2_C/MoS_2_	Er^3+^ silica fiber	1597.5	0.096	60,040	0.065	3.9	11.1 nm	[247]
	Tm^3+^ silica fiber	1901.7	0.551	22,410	0.89	20		
BP/ReS_2_	Tm^3+^: YAP	1932	3.66 × 10^5^	50	0.01	528	4 nm	[249]
Graphene/BN	Tm^3+^: YAP	1934.8	6.07 × 10^5^	188.43	19,430	2.66	monolayer/-	[236]
Graphene/WS_2_	Tm^3+^: YAP	1980.1	1.16 × 10^6^	90.36	-	1293	7 nm	[256]
WSe_2_/CuO	Tm^3+^: YAP	1986.8	7.53 × 10^5^	68.68	33,780	2320	-	[242]
WSe_2_/BN	Tm^3+^: YAP	1989.4	1.28 × 10^6^	43.51	19,170	834	-	[248]
Graphene/BN	Ho^3+^: YLF	2070	1.56 × 10^6^	52	730.36	38	monolayer/-	[257]
Graphene/Te	Er^3+^: ZBLAN	2776.8	12.18	41,700	3.58	149.3	few-layer	[238]

## Data Availability

No new data were created or analyzed in this study.

## References

[1] Kopyeva M.S., Filatova S.A., Kamynin V.A., Trikshev A.I., Kozlikina E.I., Astashov V.V., Loschenov V.B., Tsvetkov V.B. (2021). Ex-Vivo Exposure on Biological Tissues in the 2-μm Spectral Range with an All-Fiber Continuous-Wave Holmium Laser. Photonics.

[2] Xue Z., Shen F., Li J., Liu X., Wang J., Wang G., Liu K., Gao X., Tan T. (2022). A MEMS modulator-based dual-channel mid-infrared laser heterodyne radiometer for simultaneous remote sensing of atmospheric CH_4_, H_2_O and N_2_O. Opt. Express.

[3] Mingareev I., Weirauch F., Olowinsky A., Shah L., Kadwani P., Richardson M. (2012). Welding of polymers using a 2 μm thulium fiber laser. Opt. Laser Technol..

[4] Pal A., Sen R., Bremer K., Yao S., Lewis E., Sun T., Grattan K.T.V. (2012). “All-fiber” tunable laser in the 2 μm region, designed for CO_2_ detection. Appl. Opt..

[5] Li W., Chen B., Meng C., Fang W., Xiao Y., Li X., Hu Z., Xu Y., Tong L., Wang H. (2014). Ultrafast All-Optical Graphene Modulator. Nano Lett..

[6] Cao H., Ding M., Chen H., Liu C., Yu L., Zhu M., Zhao W., Guo J., Li H., Yu Z. (2024). High-Efficiency All-Optical Modulator Based on Ultra-Thin Silicon/Graphene Hybrid Waveguides. Adv. Opt. Mater..

[7] Guan X., Wang J., Zhang Y., Xu B., Luo Z., Xu H., Cai Z., Xu X., Zhang J., Xu J. (2018). Self-Q-switched and wavelength-tunable tungsten disulfide-based passively Q-switched Er:Y_2_O_3_ ceramic lasers. Photonics Res..

[8] Fecko C.J., Loparo J.J., Tokmakoff A. (2004). Generation of 45 femtosecond pulses at 3 μm with a KNbO_3_ optical parametric amplifier. Opt. Commun..

[9] Simon J. (1987). GaInAsP semiconductor laser amplifiers for single-mode fiber communications. J. Light. Technol..

[10] Yu L., Zeng Q., Wang S., Liang J., Wang J., Wang J., Luo X., Yan P., Dong F., Liu X. (2023). Mid-infrared ultrashort pulses generated from a hybrid mode-locked Er:ZBLAN fiber laser. Opt. Express.

[11] Peng R., Khaliji K., Youngblood N., Grassi R., Low T., Li M. (2017). Midinfrared Electro-optic Modulation in Few-Layer Black Phosphorus. Nano Lett..

[12] Kim J., Koo J., Lee J.H. (2017). All-fiber acousto-optic modulator based on a cladding-etched optical fiber for active mode-locking. Photonics Res..

[13] Shu H., Su Z., Huang L., Wu Z., Wang X., Zhang Z., Zhou Z. (2018). Significantly High Modulation Efficiency of Compact Graphene Modulator Based on Silicon Waveguide. Sci. Rep..

[14] Zhang Y., Wu K., Guang Z., Guo B., Qiao D., Wei Z., Yang H., Wang Q., Li K., Copner N. (2024). Advances and Challenges of Ultrafast Fiber Lasers in 2–4 µm Mid-Infrared Spectral Regions. Laser Photonics Rev..

[15] Zeng Q., Tang Z., Ouyang D., Yu L., Wang J., Luo X., Dong W., Yan P., Wang J., Wang P. (2024). Wavelength-tunable spatiotemporal mode-locking in a large-mode-area Er:ZBLAN fiber laser at 2.8 µm. Opt. Lett..

[16] Pang L., Zhao M., Zhao Q., Li L., Wang R., Wu R., Lv Y., Liu W. (2022). GaSb Film is a Saturable Absorber for Dissipative Soliton Generation in a Fiber Laser. ACS Appl. Mater. Interfaces.

[17] Ma X., Wang C., Zhang J., Wang T., Wang A., Wang S., Jia Z., Zhang B., He J., Van Smaalen S. (2022). Broadband BiOCl Nonlinear Saturable Absorber for Watt-Level Passively Q-Switched Yb:LuAG Single Crystal Fiber Laser. Adv. Opt. Mater..

[18] Zhang K., Feng M., Yang J., Li Y., Xie J., Li Y., Han D., Song F., Huang W. (2022). Niobium tellurium as a novel broadband saturable absorber for pulsed fiber lasers. J. Mater. Chem. C.

[19] Sorokin P.P., Luzzi J.J., Lankard J.R., Pettit G.D. (1964). Ruby Laser Q-Switching Elements Using Phthalocyanine Molecules in Solution. IBM J. Res. Dev..

[20] Kück S. (2001). Laser-related spectroscopy of ion-doped crystals for tunable solid-state lasers. Appl. Phys. B.

[21] Hultzsch R. (1978). Passive Q-switching of the ruby laser by means of colour centres in SrF_2_. Phys. Status Solidi A.

[22] Keller U., Miller D.A.B., Boyd G.D., Chiu T.H., Ferguson J.F., Asom M.T. (1992). Solid-state low-loss intracavity saturable absorber for Nd:YLF lasers: An antiresonant semiconductor Fabry–Perot saturable absorber. Opt. Lett..

[23] Nadimi M., Waritanant T., Major A. (2018). Passively mode-locked high power Nd:GdVO_4_ laser with direct in-band pumping at 912 nm. Laser Phys. Lett..

[24] Diebold A., Emaury F., Schriber C., Golling M., Saraceno C.J., Südmeyer T., Keller U. (2013). SESAM mode-locked Yb:CaGdAlO_4_ thin disk laser with 62 fs pulse generation. Opt. Lett..

[25] Waritanant T., Major A. (2016). High efficiency passively mode-locked Nd:YVO_4_ laser with direct in-band pumping at 914 nm. Opt. Express.

[26] Bao Q., Zhang H., Wang Y., Ni Z., Yan Y., Shen Z.X., Loh K.P., Tang D.Y. (2009). Atomic-Layer Graphene as a Saturable Absorber for Ultrafast Pulsed Lasers. Adv. Funct. Mater..

[27] Lu S.B., Miao L.L., Guo Z.N., Qi X., Zhao C.J., Zhang H., Wen S.C., Tang D.Y., Fan D.Y. (2015). Broadband nonlinear optical response in multi-layer black phosphorus: An emerging infrared and mid-infrared optical material. Opt. Express.

[28] Wang K., Wang J., Fan J., Lotya M., O’Neill A., Fox D., Feng Y., Zhang X., Jiang B., Zhao Q. (2013). Ultrafast Saturable Absorption of Two-Dimensional MoS_2_ Nanosheets. ACS Nano.

[29] Wang K., Fang J., Wang D. (2026). NiTe_2_-Based Saturable Absorber for a Passively Q-Switched Ytterbium-Doped Fiber Laser. Materials.

[30] Zhang Q., Hu Z., Hu X., Zeng G., He P., Tao L. (2025). Metallic TaS_2_: A newly found transition metal dichalcogenide for Yb-doped mode-locked fiber laser. Opt. Fiber Technol..

[31] Lee J., Kwon S.-Y., Lee J.H. (2021). Investigation on the nonlinear optical properties of V_2_C MXene at 1.9 μm. J. Mater. Chem. C.

[32] Zhang X., Chu H., Li Y., Zhao S., Li D. (2020). Diameter-selected single-walled carbon nanotubes for the passive Q-switching operation at 2 μm. Opt. Mater..

[33] Huang D., Zheng C., Huang L., Wu X., Chen L. (2018). Linear and nonlinear optical properties of ultrafine WO_3_ nanorods. Optik.

[34] Zhao C., Zhang H., Qi X., Chen Y., Wang Z., Wen S., Tang D. (2012). Ultra-short pulse generation by a topological insulator based saturable absorber. Appl. Phys. Lett..

[35] Zhang Q., Jiang X., Zhang M., Jin X., Zhang H., Zheng Z. (2020). Wideband saturable absorption in metal–organic frameworks (MOFs) for mode-locking Er- and Tm-doped fiber lasers. Nanoscale.

[36] Chen K.-Y., Wang H.-S., Su S.-P., Kuo S.-W., Lee C.-K., EL-Mahdy A.F.M. (2023). Π-Electron-Extended Porphyrin-Linked Covalent Organic Framework for a Q-Switched All-Solid-State Laser. Adv. Photonics Res..

[37] McClung F.J., Hellwarth R.W. (1962). Giant Optical Pulsations from Ruby. J. Appl. Phys..

[38] Keller U. (2003). Recent developments in compact ultrafast lasers. Nature.

[39] Set S.Y., Yaguchi H., Tanaka Y., Jablonski M. (2004). Laser Mode Locking Using a Saturable Absorber Incorporating Carbon Nanotubes. J. Light. Technol..

[40] Xia F., Wang H., Xiao D., Dubey M., Ramasubramaniam A. (2014). Two-dimensional material nanophotonics. Nat. Photonics.

[41] Li G., Bao H., Peng Y., Fu X., Liao W., Xiang C. (2024). Strain controllable band alignment and the interfacial and optical properties of tellurene/GaAs van der Waals heterostructures. Phys. Chem. Chem. Phys..

[42] Dai M., Set S.Y., Yamashita S. (2026). Ultrafast fiber lasers mode-locked by carbon nanotube and graphene saturable absorbers: Review and prospects [Invited]. Opt. Mater. Express.

[43] Koski K.J., Wessells C.D., Reed B.W., Cha J.J., Kong D., Cui Y. (2012). Chemical Intercalation of Zerovalent Metals into 2D Layered Bi_2_ Se_3_ Nanoribbons. J. Am. Chem. Soc..

[44] Li L., Yu Y., Ye G.J., Ge Q., Ou X., Wu H., Feng D., Chen X.H., Zhang Y. (2014). Black Phosphorus Field-effect Transistors. Nat. Nanotechnol..

[45] Wang F., Di Valentin C., Pacchioni G. (2011). Electronic and Structural Properties of WO_3_: A Systematic Hybrid DFT Study. J. Phys. Chem. C.

[46] Chen X., Wang Y., Shen D., Zhang M., Zhao Y., Zhou L., Qin Q., Zhang Q., He N., Wang M. (2021). First-Principles Calculation and Experimental Investigation of a Three-Atoms-Type MXene V_2_C and Its Effects on Memristive Devices. IEEE Trans. Nanotechnol..

[47] Geim A.K., Novoselov K.S. (2007). The rise of graphene. Nat. Mater..

[48] Dawlaty J.M., Shivaraman S., Chandrashekhar M., Rana F., Spencer M.G. (2008). Measurement of Ultrafast Carrier Dynamics in Epitaxial Graphene. Appl. Phys. Lett..

[49] Neto A.H.C., Guinea F., Peres N.M.R., Novoselov K.S., Geim A.K. (2009). The electronic properties of graphene. Rev. Mod. Phys..

[50] Tan W.D., Su C.Y., Knize R.J., Xie G.Q., Li L.J., Tang D.Y. (2010). Mode locking of ceramic Nd:yttrium aluminum garnet with graphene as a saturable absorber. Appl. Phys. Lett..

[51] Ma J., Xie G.Q., Lv P., Gao W.L., Yuan P., Qian L.J., Yu H.H., Zhang H.J., Wang J.Y., Tang D.Y. (2012). Graphene mode-locked femtosecond laser at 2 μm wavelength. Opt. Lett..

[52] Zhang M., Kelleher E.J.R., Torrisi F., Sun Z., Hasan T., Popa D., Wang F., Ferrari A.C., Popov S.V., Taylor J.R. (2012). Tm-doped fiber laser mode-locked by graphene-polymer composite. Opt. Express.

[53] Zen D.I.M., Saidin N., Damanhuri S.S.A., Harun S.W., Ahmad H., Ismail M.A., Dimyati K., Halder A., Paul M.C., Das S. (2013). Mode-locked thulium–bismuth codoped fiber laser using graphene saturable absorber in ring cavity. Appl. Opt..

[54] Wang Q., Chen T., Zhang B., Li M., Lu Y., Chen K.P. (2013). All-fiber passively mode-locked thulium-doped fiber ring laser using optically deposited graphene saturable absorbers. Appl. Phys. Lett..

[55] Sotor J., Sobon G., Pasternak I., Krajewska A., Strupinski W., Abramski K.M. (2013). Simultaneous mode-locking at 1565 nm and 1944 nm in fiber laser based on common graphene saturable absorber. Opt. Express.

[56] Cizmeciyan M.N., Kim J.W., Bae S., Hong B.H., Rotermund F., Sennaroglu A. (2013). Graphene mode-locked femtosecond Cr:ZnSe laser at 2500 nm. Opt. Lett..

[57] Wei C., Zhu X., Wang F., Xu Y., Balakrishnan K., Song F., Norwood R.A., Peyghambarian N. (2013). Graphene Q-switched 2.78 μm Er^3+^-doped fluoride fiber laser. Opt. Lett..

[58] Tang Y., Yu X., Li X., Yan Z., Wang Q.J. (2014). High-power thulium fiber laser Q switched with single-layer graphene. Opt. Lett..

[59] Choi S.Y., Jeong H., Hong B.H., Rotermund F., Yeom D.-I. (2014). All-fiber dissipative soliton laser with 10.2 nJ pulse energy using an evanescent field interaction with graphene saturable absorber. Laser Phys. Lett..

[60] Sobon G., Sotor J., Pasternak I., Krajewska A., Strupinski W., Abramski K.M. (2015). Multilayer graphene-based saturable absorbers with scalable modulation depth for mode-locked Er- and Tm-doped fiber lasers. Opt. Mater. Express.

[61] Sotor J., Pasternak I., Krajewska A., Strupinski W., Sobon G. (2015). Sub-90 fs a stretched-pulse mode-locked fiber laser based on a graphene saturable absorber. Opt. Express.

[62] Purdie D.G., Popa D., Wittwer V.J., Jiang Z., Bonacchini G., Torrisi F., Milana S., Lidorikis E., Ferrari A.C. (2015). Few-cycle pulses from a graphene mode-locked all-fiber laser. Appl. Phys. Lett..

[63] Jin M., Chang K., Li C., Zhang R., Li Z., Wang X., Chen K. (2022). PECVD-derived graphene saturable absorber mirror for 2.8 μm pulsed Er:ZBLAN fiber laser. J. Phys. Appl. Phys..

[64] Sobon G., Sotor J., Przewolka A., Pasternak I., Strupinski W., Abramski K. (2016). Amplification of noise-like pulses generated from a graphene-based Tm-doped all-fiber laser. Opt. Express.

[65] Chen H.-R., Tsai C.-Y., Cheng H.-M., Lin K.-H., Chen C.-H., Hsieh W.-F. (2016). High-power passively continuous-wave and Q-switching mode-locked Nd:LuVO_4_ laser by few-layer graphene-oxide films. Opt. Mater. Express.

[66] Zhu G., Zhu X., Wang F., Xu S., Li Y., Guo X., Balakrishnan K., Norwood R.A., Peyghambarian N. (2016). Graphene Mode-Locked Fiber Laser at 2.8 μm. IEEE Photonics Technol. Lett..

[67] Pawliszewska M., Martynkien T., Przewłoka A., Sotor J. (2018). Dispersion-managed Ho-doped fiber laser mode-locked with a graphene saturable absorber. Opt. Lett..

[68] Pushkin A.V., Migal E.A., Tokita S., Korostelin Y.V., Potemkin F.V. (2020). Femtosecond graphene mode-locked Fe:ZnSe laser at 4.4 µm. Opt. Lett..

[69] Lau K.Y., Zulkifli M.Z. (2021). 1.56 µm and 1.93 µm synchronized mode-locked fiber laser with graphene saturable absorber. Infrared Phys. Technol..

[70] Xu J.-L., Li X.-L., Wu Y.-Z., Hao X.-P., He J.-L., Yang K.-J. (2011). Graphene saturable absorber mirror for ultra-fast-pulse solid-state laser. Opt. Lett..

[71] Li X., Wang D.N., Hua K., Chen Q., Ge Y., Xia Q.K. (2022). Saturable absorber based on graphene for a hybrid passive mode-locked erbium-doped fiber laser. Opt. Fiber Technol..

[72] Ahmad H., Aidit S.N., Ooi S.I., Rezayi M., Tiu Z.C. (2017). Passively Q-switched and mode-locked erbium doped fiber laser based on N-doped graphene saturable absorber. Laser Phys..

[73] Popa D., Sun Z., Torrisi F., Hasan T., Wang F., Ferrari A.C. (2010). Sub 200fs pulse generation from a graphene mode-locked fiber laser. Appl. Phys. Lett..

[74] Muhammad F.D., Azis R.S., Latif A.A., Nor Asri N.A., Adnan N.N., Ahmad H. (2026). Harmonic soliton mode-locked zirconia-based erbium-doped fiber laser with graphene saturable absorber. Laser Phys..

[75] Hua K., Wang D.N. (2021). Coupling scheme for graphene saturable absorber in a linear cavity mode-locked fiber laser. Opt. Lett..

[76] Tarka J., Boguslawski J., Sobon G., Pasternak I., Przewloka A., Strupinski W., Sotor J., Abramski K.M. (2017). Power Scaling of an All-PM Fiber Er-Doped Mode-Locked Laser Based on Graphene Saturable Absorber. IEEE J. Sel. Top. Quantum Electron..

[77] Luo Z., Zhou M., Weng J., Huang G., Xu H., Ye C., Cai Z. (2010). Graphene-based passively Q-switched dual-wavelength erbium-doped fiber laser. Opt. Lett..

[78] Zhang H., Tang D.Y., Zhao L.M., Bao Q.L., Loh K.P. (2009). Large energy mode locking of an erbium-doped fiber laser with atomic layer graphene. Opt. Express.

[79] Sobon G., Sotor J., Pasternak I., Krajewska A., Strupinski W., Abramski K.M. (2015). All-polarization maintaining, graphene-based femtosecond Tm-doped all-fiber laser. Opt. Express.

[80] Li X., Yu X., Sun Z., Yan Z., Sun B., Cheng Y., Yu X., Zhang Y., Wang Q.J. (2015). High-power graphene mode-locked Tm/Ho co-doped fiber laser with evanescent field interaction. Sci. Rep..

[81] Yang G., Liu Y., Wang Z., Lou J., Wang Z., Liu Z. (2016). Broadband wavelength tunable mode-locked thulium-doped fiber laser operating in the 2 μm region by using a graphene saturable absorber on microfiber. Laser Phys. Lett..

[82] Jeong H., Choi S.Y., Kim M.H., Rotermund F., Cha Y.-H., Jeong D.-Y., Lee S.B., Lee K., Yeom D.-I. (2016). All-fiber Tm-doped soliton laser oscillator with 6 nJ pulse energy based on evanescent field interaction with monoloayer graphene saturable absorber. Opt. Express.

[83] Sotor J., Bogusławski J., Martynkien T., Mergo P., Krajewska A., Przewłoka A., StrupiŃski W., SoboŃ G. (2017). All-polarization-maintaining, stretched-pulse Tm-doped fiber laser, mode-locked by a graphene saturable absorber. Opt. Lett..

[84] Zuikafly S.N.F., Ahmad H., Nawawi W.M.F.W., Yahaya H., Ibrahim M.H., Latif A.A., Ahmad F. (2021). Graphene-chitin bio-composite polymer based mode locker at 2 micron region. Optik.

[85] Lin W., Duan X., Cui Z., Yao B., Dai T., Li X. (2016). A passively Q-switched Ho:YVO_4_ Laser at 2.05 μm with Graphene Saturable Absorber. Appl. Sci..

[86] Kawase H., Uehara H., Chen H., Yasuhara R. (2019). Passively Q-switched 2.9 μm Er:YAP single crystal laser using graphene saturable absorber. Appl. Phys. Express.

[87] Shi A., Bai Z., Qi Y., Wang Y., Huang F., Ding J., Lu Z. (2025). Utilizing of new transition metal dihalide saturable absorbent materials for ultrashort pulse lasers development. Opt. Laser Technol..

[88] Splendiani A., Sun L., Zhang Y., Li T., Kim J., Chim C.-Y., Galli G., Wang F. (2010). Emerging Photoluminescence in Monolayer MoS_2_. Nano Lett..

[89] Wang K., Feng Y., Chang C., Zhan J., Wang C., Zhao Q., Coleman J.N., Zhang L., Blau W.J., Wang J. (2014). Broadband ultrafast nonlinear absorption and nonlinear refraction of layered molybdenum dichalcogenide semiconductors. Nanoscale.

[90] Zhang H., Lu S.B., Zheng J., Du J., Wen S.C., Tang D.Y., Loh K.P. (2014). Molybdenum disulfide (MoS_2_) as a broadband saturable absorber for ultra-fast photonics. Opt. Express.

[91] Woodward R.I., Kelleher E.J.R., Howe R.C.T., Hu G., Torrisi F., Hasan T., Popov S.V., Taylor J.R. (2014). Tunable Q-switched fiber laser based on saturable edge-state absorption in few-layer molybdenum disulfide (MoS_2_). Opt. Express.

[92] Xia H., Li H., Lan C., Li C., Zhang X., Zhang S., Liu Y. (2014). Ultrafast erbium-doped fiber laser mode-locked by a CVD-grown molybdenum disulfide (MoS_2_) saturable absorber. Opt. Express.

[93] Wang S., Yu H., Zhang H., Wang A., Zhao M., Chen Y., Mei L., Wang J. (2014). Broadband Few-Layer MoS_2_ Saturable Absorbers. Adv. Mater..

[94] Du J., Wang Q., Jiang G., Xu C., Zhao C., Xiang Y., Chen Y., Wen S., Zhang H. (2014). Ytterbium-doped fiber laser passively mode locked by few-layer Molybdenum Disulfide (MoS_2_) saturable absorber functioned with evanescent field interaction. Sci. Rep..

[95] Khazaeizhad R., Kassani S.H., Jeong H., Yeom D.-I., Oh K. (2014). Mode-locking of Er-doped fiber laser using a multilayer MoS_2_ thin film as a saturable absorber in both anomalous and normal dispersion regimes. Opt. Express.

[96] Xu B., Cheng Y., Wang Y., Huang Y., Peng J., Luo Z., Xu H., Cai Z., Weng J., Moncorgé R. (2014). Passively Q-switched Nd:YAlO_3_ nanosecond laser using MoS_2_ as saturable absorber. Opt. Express.

[97] Chen B., Zhang X., Wu K., Wang H., Wang J., Chen J. (2015). Q-switched fiber laser based on transition metal dichalcogenides MoS_2_, MoSe_2_, WS_2_, and WSe_2_. Opt. Express.

[98] Tian Z., Wu K., Kong L., Yang N., Wang Y., Chen R., Hu W., Xu J., Tang Y. (2015). Mode-locked thulium fiber laser with MoS_2_. Laser Phys. Lett..

[99] Jung M., Lee J., Park J., Koo J., Jhon Y.M., Lee J.H. (2015). Mode-locked, 1.94-μm, all-fiberized laser using WS_2_-based evanescent field interaction. Opt. Express.

[100] Fan M., Li T., Zhao S., Li G., Ma H., Gao X., Kränkel C., Huber G. (2016). Watt-level passively Q-switched Er:Lu_2_O_3_ laser at 2.84 μm using MoS_2_. Opt. Lett..

[101] Liu W., Pang L., Han H., Liu M., Lei M., Fang S., Teng H., Wei Z. (2017). Tungsten disulfide saturable absorbers for 67 fs mode-locked erbium-doped fiber lasers. Opt. Express.

[102] Yin J., Li J., Chen H., Wang J., Yan P., Liu M., Liu W., Lu W., Xu Z., Zhang W. (2017). Large-area highly crystalline WSe_2_ atomic layers for ultrafast pulsed lasers. Opt. Express.

[103] Wang J., Jiang Z., Chen H., Li J., Yin J., Wang J., He T., Yan P., Ruan S. (2017). Magnetron-sputtering deposited WTe_2_ for an ultrafast thulium-doped fiber laser. Opt. Lett..

[104] Wang J., Jiang Z., Chen H., Li J., Yin J., Wang J., He T., Yan P., Ruan S. (2018). High energy soliton pulse generation by a magnetron-sputtering-deposition-grown MoTe_2_ saturable absorber. Photonics Res..

[105] Lv R., Chen Z., Liu S., Wang J., Li Y., Wang Y., Wang Y. (2019). Optical properties and applications of molybdenum disulfide/SiO_2_ saturable absorber fabricated by sol-gel technique. Opt. Express.

[106] Wang M., Zheng Y., Guo L., Chen X., Zhang H., Li D. (2019). Nonlinear Optical Properties of Zirconium Diselenide and Its Ultra-Fast Modulator Application. Nanomaterials.

[107] Sheng Q., Tang S., Ye F., Lu C., Wang G., Zhang H., Bai C., Zhang W. (2024). Generation of dual large energy pulses in Er^3+^-doped fiber lasers based on ZrTe_2_ saturable absorber via polarization manipulation. Opt. Laser Technol..

[108] Ahmad H., Kahar N.H.A., Yusoff N., Reduan S.A. (2021). Thulium-holmium doped fiber laser mode-locking with hafnium disulfide (HfS_2_) coated on D-shaped fiber. Optik.

[109] Ahmad H., Azali N.A., Yusoff N. (2022). Liquid phase exfoliation of hafnium diselenide and its role in initiating the mode-locked pulse laser at eye-safe wavelength region. Opt. Mater..

[110] Ahmad H., Ariffin N.A.M., Aidit S.N., Ooi S.I., Yusoff N., Zamzuri A.K. (2021). 1.9 μm mode-locked fiber laser based on evanescent field interaction with metallic vanadium diselenide (VSe_2_). Optik.

[111] Zhang W., Liang Y., Gan Y., Huang H., Liang G., Kang Q., Leng X., Jing Q., Wen Q. (2023). VTe_2_: Broadband Saturable Absorber for Passively Q-Switched Lasers in the Near- and Mid-Infrared Regions. ACS Appl. Mater. Interfaces.

[112] Liu W., Liu K., Feng F., Li R., Zhang W., Wang X., Liu J., Chen K. (2025). Atomic Oxygen-Passivated 2D Zr/Ta Telluride Crystals as Saturable Absorbers for High Power Mid-Infrared Pulse Generation. Adv. Funct. Mater..

[113] Sun S., Zhao Z., Fu S. (2025). Conventional and bound-state solitons generation in erbium-doped fiber lasers based on saturable absorbers of ReSe_2_. Opt. Fiber Technol..

[114] Xu N., Shang X., Yang F., Sui Z., Zhang H., Li D. (2023). Nonlinear photoresponse of PdSe_2_ nanosheets for soliton operations in passive mode-locked Er-doped fiber lasers. Infrared Phys. Technol..

[115] Cheng P.K., Ahmed S., Qiao J., Wong L.W., Yuen C.F., Saleque A.M., Ivan M.N.A.S., Hani S.U., Hossain M.I., Zhao J. (2022). Nonlinear optical properties of two-dimensional palladium ditelluride (PdTe_2_) and its application as aerosol jet printed saturable absorbers for broadband ultrafast photonics. Appl. Mater. Today.

[116] Ahmad H., Reduan S.A., Aidit S.N., Yusoff N., Maah M.J., Ismail M.F., Tiu Z.C. (2019). Ternary MoWSe_2_ alloy saturable absorber for passively Q-switched Yb-, Er- and Tm-doped fiber laser. Opt. Commun..

[117] Wang H., He X., Hao Q., Zhou Y., Li L. (2024). High average power passively Q-switched 2-μm laser with Mo_0.5_W_0.5_S_2_ as a saturable absorber. Opt. Mater..

[118] Ahmad H., Hidayah Abdul Kahar N., Yusoff N., Izzat Mohd Hanafi A., Ramli R., Wadi Harun S., Aisyah Reduan S. (2022). Passively mode-locked laser using HfSe_2_ as saturable absorber at 1.5 μm and 2.0 μm. Opt. Laser Technol..

[119] Chen J., Xie Z., Huang J., Hu Z., Zhao Y., Zheng Z., He J., Long H., Tao L. (2024). Unveiling the modulation potential: Comparison of MoS_2_ and MoSe_2_ as saturable absorbers in ultrafast fiber lasers. Opt. Laser Technol..

[120] Wang F., Sun S., Zhang X., Tan H., Zhu G., Xu W., Huang Y., Sun M., Jia Y., Li Z. (2025). Passively mode-locking fiber lasers for generating high repetition frequency pulse based on MoWSe_2_ saturable absorbers. Opt. Mater..

[121] Cao L., Li X., Zhang R., Wu D., Dai S., Peng J., Weng J., Nie Q. (2018). Tm-doped fiber laser mode-locking with MoS_2_-polyvinyl alcohol saturable absorber. Opt. Fiber Technol..

[122] Latiff A.A., Cheng X.S., Rusdi M.F.M., Paul M.C., Harun S.W., Ahmad H. (2018). Molybdenum disulfide saturable absorber for eye-safe mode-locked fiber laser generation. J. Nonlinear Opt. Phys. Mater..

[123] Wang S., Tang Y., Yang J., Zhong H., Fan D. (2019). MoS_2_ Q-switched 2.8 µm Er:ZBLAN fiber laser. Laser Phys..

[124] Dong L., Li D., Pan H., Li Y., Zhao S., Li G., Chu H. (2019). Pulse characteristics from a MoSe_2_ Q-switched Nd:GdVO_4_ laser at 1.3 µm. Appl. Opt..

[125] Lee J., Koo J., Lee J., Jhon Y.M., Lee J.H. (2017). All-fiberized, femtosecond laser at 1912 nm using a bulk-like MoSe_2_ saturable absorber. Opt. Mater. Express.

[126] Wu M., Li X., Wu K., Wu D., Dai S., Xu T., Nie Q. (2019). All-fiber 2 μm thulium-doped mode-locked fiber laser based on MoSe_2_-saturable absorber. Opt. Fiber Technol..

[127] Wang X., Han H., Liu D. (2020). Generation of Bright-Dark Soliton Pairs in Mode-Locked Fiber Laser Based on Molybdenum Diselenide. IEEE Access.

[128] Ahmad H., Ramli R., Samion M.Z., Yusoff N. (2021). Mode-locked thulium/holmium co-doped fiber laser using WTe_2_-covered tapered fiber. Optik.

[129] Zheng Z., Wang J., Yin J., Ouyang D., Ren X., Yan P., Wang J., Pei J., Lue Q., Ruan S. (2020). High-power mode-locked thulium-doped fiber laser with tungsten ditelluride as saturable absorber. Appl. Opt..

[130] Ahmad H., Albaqawi H.S., Yusoff N., Yi C.W. (2020). 56 nm Wide-Band Tunable Q-Switched Erbium Doped Fiber Laser with Tungsten Ditelluride (WTe_2_) Saturable Absorber. Sci. Rep..

[131] Yu H., Zheng X., Yin K., Cheng X., Jiang T. (2017). All-fiber thulium/holmium-doped mode-locked laser by tungsten disulfide saturable absorber. Laser Phys..

[132] Luan C., Yang K., Zhao J., Zhao S., Song L., Li T., Chu H., Qiao J., Wang C., Li Z. (2016). WS_2_ as a saturable absorber for Q-switched 2 micron lasers. Opt. Lett..

[133] Chen S., Wang F., Kuang F., Kang S., Liang H., Zheng L., Guan L., Wu Q. (2022). Femtosecond Pulsed Fiber Laser by an Optical Device Based on NaOH-LPE Prepared WSe_2_ Saturable Absorber. Nanomaterials.

[134] Zou D., Yan M., Chai L., Song Y., Hu M. (2022). (INVITED)A diverse set of soliton molecules generation in a passively mode-locked Er-doped fiber laser with a saturable absorber of WSe_2_ nanofilm. Results Opt..

[135] Zheng J., Zong M., Ye K., Feng X., Liu J., Liu J., Liu D., Liu Z. (2025). Monolayer WSe_2_ Film as Saturable Absorbers for Mid-Infrared Passive Q-Switching. Microw. Opt. Technol. Lett..

[136] Ge Y., Zhu Z., Xu Y., Chen Y., Chen S., Liang Z., Song Y., Zou Y., Zeng H., Xu S. (2018). Broadband Nonlinear Photoresponse of 2D TiS_2_ for Ultrashort Pulse Generation and All-Optical Thresholding Devices. Adv. Opt. Mater..

[137] He Y., Wang L., Li S., Tang Y., Lu C., Zhang W., Wang G., Bai C., Li Z., Zhang H. (2024). Correlation between soliton state and power in erbium-doped fiber lasers based on PtS_2_ saturable absorbers. Opt. Mater..

[138] Jiang S., Wei C., Zheng L., Zhou H., Liu W., Zhang J., Zhang H., Liu Y. (2022). PtSe_2_ as a Wideband Saturable Absorber for Passively Q-Switched High-Power Mid-Infrared Fiber Laser. IEEE Photonics Technol. Lett..

[139] Wang P., Zhang H., Yin Y., Ouyang Q., Chen Y., Lewis E., Farrell G., Tokurakawa M., Wadi Harun S., Wang C. (2020). NiS_2_ as a broadband saturable absorber for ultrafast pulse lasers. Opt. Laser Technol..

[140] He J., Lu H., Tao L., Zhao Y., Zheng Z., Zhou B. (2022). Novel two-dimensional semi-metallic NiTe_2_ based saturable absorber for ultrafast mode-locked fiber laser. Infrared Phys. Technol..

[141] Hu Z., Hu X., He P., Chen J., Huang J., Xie Z., Zhao Y., Tao L., Hao M., He J. (2023). NbS_2_-nanosheet-based saturable absorber for 1.5 µm and 2 µm ultrafast fiber lasers. Photonics Nanostruct.-Fundam. Appl..

[142] Wang J., Chen H., Jiang Z., Yin J., Wang J., Zhang M., He T., Li J., Yan P., Ruan S. (2018). Mode-locked thulium-doped fiber laser with chemical vapor deposited molybdenum ditelluride. Opt. Lett..

[143] Debnath P.C., Kim H., Yim J.H., Ha S., Ryu S., Min T., Yoo Y., Yeom D.-I. (2026). Phase-Engineered MoTe_2_ Saturable Absorbers for Diverse Fiber Pulse Laser Operations. J. Light. Technol..

[144] Wang Y., Huang G., Mu H., Lin S., Chen J., Xiao S., Bao Q., He J. (2015). Ultrafast recovery time and broadband saturable absorption properties of black phosphorus suspension. Appl. Phys. Lett..

[145] Chen Y., Jiang G., Chen S., Guo Z., Yu X., Zhao C., Zhang H., Bao Q., Wen S., Tang D. (2015). Mechanically exfoliated black phosphorus as a new saturable absorber for both Q-switching and Mode-locking laser operation. Opt. Express.

[146] Sotor J., Sobon G., Kowalczyk M., Macherzynski W., Paletko P., Abramski K.M. (2015). Ultrafast thulium-doped fiber laser mode locked with black phosphorus. Opt. Lett..

[147] Qin Z., Xie G., Zhang H., Zhao C., Yuan P., Wen S., Qian L. (2015). Black phosphorus as saturable absorber for the Q-switched Er:ZBLAN fiber laser at 2.8 μm. Opt. Express.

[148] Luo Z.-C., Liu M., Guo Z.-N., Jiang X.-F., Luo A.-P., Zhao C.-J., Yu X.-F., Xu W.-C., Zhang H. (2015). Microfiber-based few-layer black phosphorus saturable absorber for ultra-fast fiber laser. Opt. Express.

[149] Mu H., Lin S., Wang Z., Xiao S., Li P., Chen Y., Zhang H., Lau S.P., Pan C., Fan D. (2015). Black Phosphorus-Polymer Composites for Pulsed Lasers. Adv. Opt. Mater..

[150] Zhang R., Zhang Y., Yu H., Zhang H., Yang R., Liu Z., Wang J. (2015). Broadband black phosphorus optical modulator in visible to mid-infrared spectral range. Adv. Opt. Mater..

[151] Ma J., Lu S., Guo Z., Xu X., Zhang H., Tang D., Fan D. (2015). Few-layer black phosphorus based saturable absorber mirror for pulsed solid-state lasers. Opt. Express.

[152] Lu D., Pan Z., Zhang R., Xu T., Yang R., Yang B., Liu Z., Yu H., Zhang H., Wang J. (2016). Passively Q-switched ytterbium-doped ScBO_3_ laser with black phosphorus saturable absorber. Opt. Eng..

[153] Su X., Wang Y., Zhang B., Zhao R., Yang K., He J., Hu Q., Jia Z., Tao X. (2016). Femtosecond solid-state laser based on a few-layered black phosphorus saturable absorber. Opt. Lett..

[154] Zhang B., Lou F., Zhao R., He J., Li J., Su X., Ning J., Yang K. (2015). Exfoliated layers of black phosphorus as saturable absorber for ultrafast solid-state laser. Opt. Lett..

[155] Xie Y., Kong L., Qin Z., Xie G., Zhang J. (2016). Black phosphorus-based saturable absorber for Q-switched Tm:YAG ceramic laser. Opt. Eng..

[156] Wang Z., Zhao R., He J., Zhang B., Ning J., Wang Y., Su X., Hou J., Lou F., Yang K. (2016). Multi-layered black phosphorus as saturable absorber for pulsed Cr:ZnSe laser at 2.4 μm. Opt. Express.

[157] Zhang Q., Jin X., Hu G., Zhang M., Zheng Z., Hasan T. (2020). Sub-150 fs dispersion-managed soliton generation from an all-fiber Tm-doped laser with BP-SA. Opt. Express.

[158] Qin Z., Hai T., Xie G., Ma J., Yuan P., Qian L., Li L., Zhao L., Shen D. (2018). Black phosphorus Q-switched and mode-locked mid-infrared Er:ZBLAN fiber laser at 3.5 μm wavelength. Opt. Express.

[159] Jin X., Hu G., Zhang M., Hu Y., Albrow-Owen T., Howe R.C.T., Wu T.-C., Wu Q., Zheng Z., Hasan T. (2018). 102 fs pulse generation from a long-term stable, inkjet-printed black phosphorus-mode-locked fiber laser. Opt. Express.

[160] Zhang H., He J., Wang Z., Hou J., Zhang B., Zhao R., Han K., Yang K., Nie H., Sun X. (2016). Dual-wavelength, passively Q-switched Tm:YAP laser with black phosphorus saturable absorber. Opt. Mater. Express.

[161] Pawliszewska M., Ge Y., Li Z., Zhang H., Sotor J. (2017). Fundamental and harmonic mode-locking at 2.1 μm with black phosphorus saturable absorber. Opt. Express.

[162] Sun X., Nie H., He J., Zhao R., Su X., Wang Y., Zhang B., Wang R., Yang K. (2017). Passively mode-locked 1.34 μm bulk laser based on few-layer black phosphorus saturable absorber. Opt. Express.

[163] Liu J., Chen Y., Li Y., Zhang H., Zheng S., Xu S. (2018). Switchable dual-wavelength Q-switched fiber laser using multilayer black phosphorus as a saturable absorber. Photonics Res..

[164] Park K., Lee J., Lee Y.T., Choi W., Lee J.H., Song Y. (2015). Black phosphorus saturable absorber for ultrafast mode-locked pulse laser via evanescent field interaction. Ann. Phys..

[165] Song Y., Chen S., Zhang Q., Li L., Zhao L., Zhang H., Tang D. (2016). Vector soliton fiber laser passively mode locked by few layer black phosphorus-based optical saturable absorber. Opt. Express.

[166] Ahmed M.H.M., Latiff A.A., Arof H., Harun S.W. (2016). Ultrafast erbium-doped fiber laser mode-locked with a black phosphorus saturable absorber. Laser Phys. Lett..

[167] Ismail E.I., Kadir N.A., Latiff A.A., Ahmad H., Harun S.W. (2016). Black phosphorus crystal as a saturable absorber for both a Q-switched and mode-locked erbium-doped fiber laser. RSC Adv..

[168] Liu S., Zhang Y., Li L., Wang Y., Lv R., Wang X., Chen Z., Wei L. (2018). Er-doped Q-switched fiber laser with a black phosphorus/polymethyl methacrylate saturable absorber. Appl. Opt..

[169] Markom A.M., Tan S.J., Muhammad A.R., Paul M.C., Dhar A., Das S., Latiff A.A., Harun S.W. (2020). Dark pulse mode-locked fibre laser with zirconia-based erbium-doped fibre (Zr-EDF) and Black phosphorus saturable absorber. Optik.

[170] Guo L., Li T., Zhang S., Wang M., Yang K., Fan M., Zhao S., Li M. (2018). Black phosphorus saturable absorber for Q-switched Er:YAG laser at 1645 nm. Opt. Laser Technol..

[171] Yu H., Zheng X., Yin K., Cheng X., Jiang T. (2015). Thulium/holmium-doped fiber laser passively mode locked by black phosphorus nanoplatelets-based saturable absorber. Appl. Opt..

[172] Latiff A.A., Rusdi M.F.M., Jusoh Z., Yasin M., Ahmad H., Harun S.W. (2016). 1941 nm Q-switched thulium-doped fiber laser with a multi-layer black phosphorus saturable absorber. Optoelectron. Adv. Mater.-Rapid Commun..

[173] Wang Y., Li J., Han L., Lu R., Hu Y., Li Z., Liu Y. (2016). Q-switched Tm^3+^-doped fiber laser with a micro-fiber based black phosphorus saturable absorber. Laser Phys..

[174] Liu J., Liu J., Guo Z., Zhang H., Ma W., Wang J., Su L. (2016). Dual-wavelength Q-switched Er:SrF_2_ laser with a black phosphorus absorber in the mid-infrared region. Opt. Express.

[175] Li C., Liu J., Guo Z., Zhang H., Ma W., Wang J., Xu X., Su L. (2018). Black phosphorus saturable absorber for a diode-pumped passively Q-switched Er:CaF_2_ mid-infrared laser. Opt. Commun..

[176] Ostojic G.N., Zaric S., Kono J., Strano M.S., Moore V.C., Hauge R.H., Smalley R.E. (2004). Interband Recombination Dynamics in Resonantly Excited Single-Walled Carbon Nanotubes. Phys. Rev. Lett..

[177] Rozhin A.G., Sakakibara Y., Namiki S., Tokumoto M., Kataura H., Achiba Y. (2006). Sub-200-fs pulsed erbium-doped fiber laser using a carbon nanotube-polyvinylalcohol mode locker. Appl. Phys. Lett..

[178] Solodyankin M.A., Obraztsova E.D., Lobach A.S., Chernov A.I., Tausenev A.V., Konov V.I., Dianov E.M. (2008). Mode-locked 1.93 μm thulium fiber laser with a carbon nanotube absorber. Opt. Lett..

[179] Zhang Y., Petrov V., Griebner U., Zhang X., Choi S.Y., Gwak J.Y., Rotermund F., Mateos X., Yu H., Zhang H. (2014). 90-fs diode-pumped Yb:CLNGG laser mode-locked using single-walled carbon nanotube saturable absorber. Opt. Express.

[180] Tang C.Y., Chai Y., Long H., Tao L., Zeng L.H., Tsang Y.H., Zhang L., Lin X. (2015). High-power passively mode-locked Nd:YVO_4_ laser using SWCNT saturable absorber fabricated by dip coating method. Opt. Express.

[181] Wei J., Li P., Yu L., Ruan S., Li K., Yan P., Wang J., Wang J., Guo C., Liu W. (2022). Mode-locked fiber laser of 3.5 μm using a single-walled carbon nanotube saturable absorber mirror. Chin. Opt. Lett..

[182] Zhang H., Liu C.-X., Qi X.-L., Dai X., Fang Z., Zhang S.-C. (2009). Topological insulators in Bi_2_Se_3_, Bi_2_Te_3_ and Sb_2_Te_3_ with a single Dirac cone on the surface. Nat. Phys..

[183] Yu H., Zhang H., Wang Y., Zhao C., Wang B., Wen S., Zhang H., Wang J. (2013). Topological insulator as an optical modulator for pulsed solid-state lasers. Laser Photonics Rev..

[184] Jung M., Lee J., Koo J., Park J., Song Y.-W., Lee K., Lee S., Lee J.H. (2014). A femtosecond pulse fiber laser at 1935 nm using a bulk-structured Bi_2_Te_3_ topological insulator. Opt. Express.

[185] Yin K., Zhang B., Li L., Jiang T., Zhou X., Hou J. (2015). Soliton mode-locked fiber laser based on topological insulator Bi_2_Te_3_ nanosheets at 2 μm. Photonics Res..

[186] Liu W., Pang L., Han H., Tian W., Chen H., Lei M., Yan P., Wei Z. (2016). 70-fs mode-locked erbium-doped fiber laser with topological insulator. Sci. Rep..

[187] Zhu C., Wang F., Meng Y., Yuan X., Xiu F., Luo H., Wang Y., Li J., Lv X., He L. (2017). A robust and tuneable mid-infrared optical switch enabled by bulk Dirac fermions. Nat. Commun..

[188] Hou S., Lu C., Ma Z., Kang L., Lin H., Zhang M., Yan P. (2022). Broadband GaSb saturable absorber for pulse generation from C-band to mid-infrared. J. Lumin..

[189] Bai X., Mou C., Xu L., Wang S., Pu S., Zeng X. (2016). Passively Q-switched erbium-doped fiber laser using Fe_3_O_4_-nanoparticle saturable absorber. Appl. Phys. Express.

[190] Mohd Rusdi M.F., Latiff A.A., Paul M.C., Das S., Dhar A., Ahmad H., Harun S.W. (2017). Titanium Dioxide (TiO_2_) film as a new saturable absorber for generating mode-locked Thulium-Holmium doped all-fiber laser. Opt. Laser Technol..

[191] Yang J., Hu J., Luo H., Li J., Liu J., Li X., Liu Y. (2020). Fe_3_O_4_ nanoparticles as a saturable absorber for a tunable Q-switched dysprosium laser around 3 μm. Photonics Res..

[192] Ahmed M.H.M., Mohd Yusoff N., Zainol Abidin N.H., Lee H.K., Alresheedi M.T., Abas A.F., Goh C.S., Mahdi M.A. (2022). Ultrashort pulse thulium-doped fiber laser with molybdenum trioxide on tapered fiber. Optik.

[193] Jhon Y.I., Koo J., Anasori B., Seo M., Lee J.H., Gogotsi Y., Jhon Y.M. (2017). Metallic MXene Saturable Absorber for Femtosecond Mode-Locked Lasers. Adv. Mater..

[194] Jiang X., Liu S., Liang W., Luo S., He Z., Ge Y., Wang H., Cao R., Zhang F., Wen Q. (2018). Broadband Nonlinear Photonics in Few-Layer MXene Ti_3_C_2_T_x_ (T = F, O, or OH). Laser Photonics Rev..

[195] Yang Q., Zhang F., Zhang N., Zhang H. (2019). Few-layer MXene Ti_3_C_2_T_x_ (T = F, O, or OH) saturable absorber for visible bulk laser. Opt. Mater. Express.

[196] Wang Z., Li H., Luo M., Chen T., Xia X., Chen H., Ma C., Guo J., He Z., Song Y. (2020). MXene Photonic Devices for Near-Infrared to Mid-Infrared Ultrashort Pulse Generation. ACS Appl. Nano Mater..

[197] Jhon Y.I., Lee J., Jhon Y.M., Lee J.H. (2021). Ultrafast mode-locking in highly stacked Ti_3_ C_2_ T_x_ MXenes for 1.9-μm infrared femtosecond pulsed lasers. Nanophotonics.

[198] Gao L., Chen H., Zhang F., Mei S., Zhang Y., Bao W., Ma C., Yin P., Guo J., Jiang X. (2020). Ultrafast Relaxation Dynamics and Nonlinear Response of Few-Layer Niobium Carbide MXene. Small Methods.

[199] Ahmad H., Ramli R., Yusoff N., Reduan S.A., Zamzuri A.K., Thambiratnam K. (2021). Performance of Nb_2_C MXene coated on tapered fiber as saturable absorber for the generation of Mode-Locked Erbium-Doped fiber laser. Infrared Phys. Technol..

[200] Chernysheva M.A., Krylov A.A., Kryukov P.G., Arutyunyan N.R., Pozharov A.S., Obraztsova E.D., Dianov E.M. (2012). Thulium-doped mode-locked all-fiber laser based on NALM and carbon nanotube saturable absorber. Opt. Express.

[201] Chernysheva M., Bednyakova A., Al Araimi M., Howe R.C.T., Hu G., Hasan T., Gambetta A., Galzerano G., Rümmeli M., Rozhin A. (2017). Double-Wall Carbon Nanotube Hybrid Mode-Locker in Tm-doped Fibre Laser: A Novel Mechanism for Robust Bound-State Solitons Generation. Sci. Rep..

[202] Sobon G., Duzynska A., Świniarski M., Judek J., Sotor J., Zdrojek M. (2017). CNT-based saturable absorbers with scalable modulation depth for Thulium-doped fiber lasers operating at 1.9 μm. Sci. Rep..

[203] Wang J., Liang X., Hu G., Zheng Z., Lin S., Ouyang D., Wu X., Yan P., Ruan S., Sun Z. (2016). 152 fs nanotube-mode-locked thulium-doped all-fiber laser. Sci. Rep..

[204] Wang Q., Chen T., Li M., Zhang B., Lu Y., Chen K.P. (2013). All-fiber ultrafast thulium-doped fiber ring laser with dissipative soliton and noise-like output in normal dispersion by single-wall carbon nanotubes. Appl. Phys. Lett..

[205] Niu C., Wang Z., Zhang J., Yu T., Zhou J., Li N., Qin G., Ning D., Zhang F., Feng D. (2016). Tunable dual-wavelength passively mode-locked thulium-doped fiber laser using carbon nanotube. Opt. Eng..

[206] Chen Y., Zhai J., Xu X., Li L., Wang J., Zhang M., Ruan S., Tang Z. (2017). Mode-locked thulium-doped fiber laser based on 0.3 nm diameter single-walled carbon nanotubes at 1.95 μm. Chin. Opt. Lett..

[207] Meng Y., Li Y., Xu Y., Wang F. (2017). Carbon Nanotube Mode-Locked Thulium Fiber Laser with 200 nm Tuning Range. Sci. Rep..

[208] Pawliszewska M., Dużyńska A., Zdrojek M., Sotor J. (2019). Metallic carbon nanotube-based saturable absorbers for holmium-doped fiber lasers. Opt. Express.

[209] Lee J., Lee J.H. (2018). Femtosecond Tm–Ho co-doped fiber laser using a bulk-structured Bi_2_ Se_3_ topological insulator. Chin. Phys. B.

[210] Lee J., Kim T., Lee J.H. (2020). Investigation into nonlinear optical absorption property of CoSb_3_ skutterudite in the 2 μm spectral region. Opt. Laser Technol..

[211] Haris H., Harun S.W., Muhammad A.R., Anyi C.L., Tan S.J., Ahmad F., Nor R.M., Zulkepely N.R., Arof H. (2017). Passively Q-switched Erbium-doped and Ytterbium-doped fibre lasers with topological insulator bismuth selenide (Bi_2_Se_3_) as saturable absorber. Opt. Laser Technol..

[212] Guo B., Yao Y., Yang Y.-F., Yuan Y.-J., Jin L., Yan B., Zhang J.-Y. (2015). Dual-wavelength rectangular pulse erbium-doped fiber laser based on topological insulator saturable absorber. Photonics Res..

[213] Haris H., Arof H., Muhammad A.R., Anyi C.L., Tan S.J., Kasim N., Harun S.W. (2019). Passively Q-switched and mode-locked Erbium-doped fiber laser with topological insulator Bismuth Selenide (Bi_2_Se_3_) as saturable absorber at C-band region. Opt. Fiber Technol..

[214] Ma X., Chen W., Tong L., Liu S., Dai W., Ye S., Zheng Z., Wang Y., Zhou Y., Zhang W. (2021). Experimental demonstration of harmonic mode-locking in Sb_2_Se_3_-based thulium-doped fiber laser. Opt. Laser Technol..

[215] Sotor J., Sobon G., Macherzynski W., Abramski K.M. (2014). Harmonically mode-locked Er-doped fiber laser based on a Sb_2_Te_3_ topological insulator saturable absorber. Laser Phys. Lett..

[216] Chen H.-R., Tsai C.-Y., Cheng H.-M., Lin K.-H., Yen P.-H., Chen C.-H., Hsieh W.-F. (2016). High-quality and Large-size Topological Insulator Bi_2_Te_3_-Gold Saturable Absorber Mirror for Mode-Locking Fiber Laser. Sci. Rep..

[217] Gao P., Huang H., Wang X., Liu H., Huang J., Weng W., Dai S., Li J., Lin W. (2018). Passively Q-switched solid-state Tm:YAG laser using topological insulator Bi_2_Te_3_ as a saturable absorber. Appl. Opt..

[218] Ahmad H., Kamaruzzaman K., Samion M.Z. (2024). Mode-Locking and Q-Switching in Holmium Doped Fiber Laser Using Topological Insulator (Sb_2_Te_3_) as Saturable Absorber. IEEE J. Quantum Electron..

[219] Yan P., Lin R., Ruan S., Liu A., Chen H., Zheng Y., Chen S., Guo C., Hu J. (2015). A practical topological insulator saturable absorber for mode-locked fiber laser. Sci. Rep..

[220] Zhao C., Zou Y., Chen Y., Wang Z., Lu S., Zhang H., Wen S., Tang D. (2012). Wavelength-tunable picosecond soliton fiber laser with Topological Insulator: Bi_2_Se_3_ as a mode locker. Opt. Express.

[221] Zhang M., Kang L., Ma Z., Yan P., Hou S. (2022). Te Film as a Saturable Absorber for the Mid-Infrared Er^3+^-Doped ZBLAN Fiber Laser. Front. Phys..

[222] Ahmed M.H.M., Mohd Yusoff N., Che Abdullah C.A., Alresheedi M.T., Rosli N.S., Talib Z.A., Mahdi M.A. (2021). Nanosized titanium dioxide saturable absorber for soliton mode-locked thulium-doped fiber laser. Results Phys..

[223] Ahmad H., Samion M.Z., Yusoff N. (2018). Soliton mode-locked thulium-doped fiber laser with cobalt oxide saturable absorber. Opt. Fiber Technol..

[224] Ahmad H., Samion M.Z., Kamely A.A., Ismail M.F. (2019). Mode-locked thulium doped fiber laser with zinc oxide saturable absorber for 2 μm operation. Infrared Phys. Technol..

[225] Ahmad H., Ismail N.N., Aidit S.N., Yusoff N., Ramli R., Zamzuri A.K. (2020). Soliton passively mode-locked pulses generation in thulium-holmium doped fiber laser (THDFL) with molybdenum oxide saturable absorber. Opt. Fiber Technol..

[226] Du R., Shi A., Lian Y., Fang H., Qi Y., Bai Z., Wang Y., Huang F., Ding J., Lu Z. (2025). Nonlinear optical response of BiVO_4_ and its application for ultrafast pulsed fiber lasers. Opt. Express.

[227] Ahmad H., Abdul Kahar N.H., Ramli R., Yusoff N., Reduan S.A., Ismail M.F., Lim K.S., Chong W.Y., Yasin M. (2021). The performance of Ti_2_C MXene and Ti_2_AlC MAX Phase as saturable absorbers for passively mode-locked fiber laser. Opt. Fiber Technol..

[228] Ahmad H., Ramli R., Reduan S.A., Ismail M.F., Yasin M. (2021). Mode-locked thulium/holmium-doped fiber laser with vanadium carbide deposited on tapered fiber. Opt. Fiber Technol..

[229] Stankovich S., Dikin D.A., Dommett G.H.B., Kohlhaas K.M., Zimney E.J., Stach E.A., Piner R.D., Nguyen S.T., Ruoff R.S. (2006). Graphene-based composite materials. Nature.

[230] Chen H., Yin J., Yang J., Zhang X., Liu M., Jiang Z., Wang J., Sun Z., Guo T., Liu W. (2017). Transition-metal dichalcogenides heterostructure saturable absorbers for ultrafast photonics. Opt. Lett..

[231] Quan C., Lu C., He C., Xu X., Huang Y., Zhao Q., Xu X. (2019). Band Alignment of MoTe_2_ /MoS_2_ Nanocomposite Films for Enhanced Nonlinear Optical Performance. Adv. Mater. Interfaces.

[232] Sun X., Zhang B., Li Y., Luo X., Li G., Chen Y., Zhang C., He J. (2018). Tunable Ultrafast Nonlinear Optical Properties of Graphene/MoS_2_ van der Waals Heterostructures and Their Application in Solid-State Bulk Lasers. ACS Nano.

[233] Qiao H., Yuan J., Xu Z., Chen C., Lin S., Wang Y., Song J., Liu Y., Khan Q., Hoh H.Y. (2015). Broadband Photodetectors Based on Graphene–Bi_2_Te_3_ Heterostructure. ACS Nano.

[234] Long H., Hu J.-W., Wu F.-G., Dong H.-F. (2020). Ultrafast pulse lasers based on two-dimensional nanomaterial heterostructures as saturable absorber. Acta Phys. Sin..

[235] Mu H., Wang Z., Yuan J., Xiao S., Chen C., Chen Y., Chen Y., Song J., Wang Y., Xue Y. (2015). Graphene–Bi_2_Te_3_ Heterostructure as Saturable Absorber for Short Pulse Generation. ACS Photonics.

[236] Gao L., Ding Y., Zhai X., Min H., Liu G., Lan R., Shen Y. (2024). Passively Q-switched 2 μm laser based on graphene/BN heterostructure as saturable absorber. Opt. Laser Technol..

[237] Shao J., Yao G., Wu X., Lin K., Zhang S., Cheng X., Zhong D., Liu C., Liu C., Wang F. (2025). Robust mode-locking in all-fiber ultrafast laser by nanocavity of two-dimensional heterostructure. Light Sci. Appl..

[238] Wang B., Liu P., Zhang S., Cai E., Zhang L., Tian Y., Zhang J. (2025). Passively Q-Switched and Mode-Locked Laser Generation in 3 μm Fluoride Fibers Based on Tellurium/Graphene Heterojunction Saturable Absorber. J. Nonlinear Opt. Phys. Mater..

[239] Liu W., Zhu Y.-N., Liu M., Wen B., Fang S., Teng H., Lei M., Liu L.-M., Wei Z. (2018). Optical properties and applications for MoS_2_-Sb_2_Te_3_-MoS_2_ heterostructure materials. Photonics Res..

[240] Liu W.J., Liu M.L., Liu B., Quhe R.G., Lei M., Fang S.B., Teng H., Wei Z.Y. (2019). Nonlinear optical properties of MoS_2_-WS_2_ heterostructure in fiber lasers. Opt. Express.

[241] Pang L., Jiang L., Zhao M., Zhang J., Zhao Q., Li L., Wu R., Lv Y., Liu W. (2025). Ti_3_C_2_T_x_/CuO heterojunction for ultrafast photonics. J. Mater. Sci. Technol..

[242] Yang Y., Gao L., Han Y., Gao Q., Lan R., Shen Y. (2024). Passively Q-switched Tm:YAP laser based on WSe_2_/CuO heterojunction saturable absorber. Appl. Phys. B.

[243] Wang M., Xia W., Wang J., Zhang X., Guo Y., Li G., Chen P., Song P., Zhao G. (2026). Preparation of a 2D WS_2_ /MoS_2_ heterostructure *via* S-vacancy doping and its application in ultrafast laser modulation. J. Mater. Chem. C.

[244] Liu J., Chen S., He J., Tao L., Zhao Y. (2023). TiO_2_@Ti_3_C_2_T_x_ Heterostructure as an Environmentally Stable Saturable Absorber for Ultrafast Photonics. Opt. Mater..

[245] Song Y., You K., Zhao J., Huang D., Chen Y., Xing C., Zhang H. (2020). A nano-lateral heterojunction of selenium-coated tellurium for infrared-band soliton fiber lasers. Nanoscale.

[246] Pang Q., Zhu X., Shi L., Xu B., Weng R., Wang J., Zhou C., Fan M., Tang W., Xia W. (2024). Generation of Bright-Dark soliton pairs in mode-locked fiber laser based on WSe_2_/MoSe_2_ heterojunction. Infrared Phys. Technol..

[247] Wu Q., Peng L., Zhao J., Sun K., Chen S., Huang W. (2025). MXene Nb_2_C/MoS_2_ heterostructure: Nonlinear optical properties and a new broadband saturable absorber for ultrafast photonics. Mater. Today Phys..

[248] Gao L., Zhai X., Jiang L., Sui Q., Niu D., Zhang Q., Lan R., Shen Y. (2024). WSe_2_/BN heterostructure as saturable absorber for a diode-pumped passively Q-switched 2 µm solid-state laser. Opt. Express.

[249] Li H., Tang W., Shan Y., Wang J., Jiang K., Fan M., Chen T., Zhou C., Xia W. (2025). Nonlinear saturable absorption properties of BP/ReS_2_ heterojunction and its application in 2 μm all-solid-state lasers. Front. Optoelectron..

[250] Zhang L., Liu J., Li J., Wang Z., Wang Y., Ge Y., Dong W., Xu N., He T., Zhang H. (2020). Site-Selective Bi_2_Te_3_–FeTe_2_ Heterostructure as a Broadband Saturable Absorber for Ultrafast Photonics. Laser Photonics Rev..

[251] Liu S., Li Z., Ge Y., Wang H., Yue R., Jiang X., Li J., Wen Q., Zhang H. (2017). Graphene/phosphorene nano-heterojunction: Facile synthesis, nonlinear optics, and ultrafast photonics applications with enhanced performance. Photonics Res..

[252] Li Q., Li H., Han M., Chang H., Shu X. (2022). Low threshold Q-switched pulses based on a WTe_2_-graphene saturable absorber. Opt. Mater. Express.

[253] Mu H., Tuo M., Xu C., Bao X., Xiao S., Sun T., Li L., Zhao L., Li S., Ren W. (2019). Graphene and Mo_2_C vertical heterostructure for femtosecond mode-locked lasers [Invited]. Opt. Mater. Express.

[254] Jiang Y., Miao L., Jiang G., Chen Y., Qi X., Jiang X., Zhang H., Wen S. (2015). Broadband and enhanced nonlinear optical response of MoS_2_/graphene nanocomposites for ultrafast photonics applications. Sci. Rep..

[255] Du W., Li H., Lan C., Li C., Li J., Wang Z., Liu Y. (2020). Graphene/WS_2_ heterostructure saturable absorbers for ultrashort pulse generation in L-band passively mode-locked fiber lasers. Opt. Express.

[256] Zhang X., Shi Y., Zong T., Liu B., Mu Y., Liu L. (2023). Passive Q-Switched Operation of Tm:YAP Laser with Graphene/WS_2_ Heterostructure Saturable Absorber. J. Russ. Laser Res..

[257] Zhai X., Ding Y., Min H., Gao L., Liu G., Lan R., Shen Y. (2023). An infrared passively Q-switched laser based on graphene/BN heterojunction. Infrared Phys. Technol..

[258] Sun B., Pang J., Cheng Q., Zhang S., Li Y., Zhang C., Sun D., Ibarlucea B., Li Y., Chen D. (2021). Synthesis of Wafer-Scale Graphene with Chemical Vapor Deposition for Electronic Device Applications. Adv. Mater. Technol..

[259] Zhang Y.-H., Smith D.J. (2021). Heterovalent semiconductor structures and devices grown by molecular beam epitaxy. J. Vac. Sci. Technol. A.

[260] Li L., Zhou M., Jin L., Liu L., Mo Y., Li X., Mo Z., Liu Z., You S., Zhu H. (2019). Research Progress of the Liquid-Phase Exfoliation and Stable Dispersion Mechanism and Method of Graphene. Front. Mater..

[261] Adalati R., Kumar A., Malik G., Chandra R. (2020). Transition metal nitride nanoflake thin film grown by DC-magnetron sputtering for high-performance supercapacitor applications. AIP Conf. Proc..

[262] Deng Y., Zhu C., Wang Y., Wang X., Zhao X., Wu Y., Tang B., Duan R., Zhou K., Liu Z. (2022). Lithography-free, high-density MoTe_2_ nanoribbon arrays. Mater. Today.

